# T‐circle vector strategy increases NHEJ‐mediated site‐specific integration in soybean

**DOI:** 10.1111/pbi.14311

**Published:** 2024-02-19

**Authors:** Xudong Ye, John Bradley, Larry Gilbertson

**Affiliations:** ^1^ Bayer Crop Science Chesterfield Missouri USA

**Keywords:** Agrobacterium, T‐circle, CRISPR/Cas12a, soybean transformation, non‐homologous end joining, site‐specific integration

During *Agrobacterium*‐mediated transformation, linear single‐stranded DNA (T‐strand) carrying genes of interest (GOIs) from a right border (RB) to a left border (LB) in the transfer DNA (T‐DNA) of a binary vector is transferred into plant cells and randomly integrated into the plant genome (Gelvin, 2021). Circular T‐DNA molecules (T‐circles) in plant cells have been reported (Bakkeren *et al*., 1989) and suggested to be not integrated (Gelvin, 2021; Singer *et al*., 2012).

We used a non‐homologous end joining (NHEJ) repair pathway (Song *et al*., 2021) for site‐specific integration (SSI) to insert the entire T‐DNA into the DT5.1 target site in the soybean genome. The *LbCas12a* (Zetsche *et al*., 2015) and gRNA expression cassettes were placed in a conventional T‐DNA (Figure 1a) to deliver genes into soybean plants by *Agrobacterium*. The SSI frequency of single copy, backbone‐free and perfect genetic element (SC‐BF‐PGE) events was 0.3% (Table S1).

**Figure 1:**
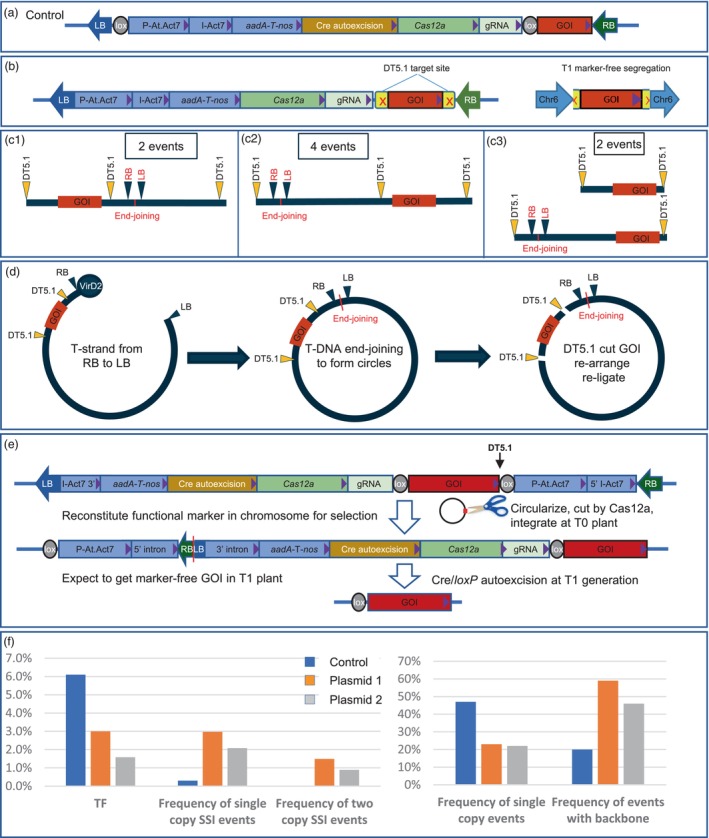
T‐DNA structures in *Agrobacterium* binary vectors and transgenic soybean plants and the frequencies of transgenic events. (a) T‐DNA configuration from the control binary vector with intact marker gene. (b) T‐DNA structure with two DT5.1 target sites flanking the GOI (left) and the expected GOI integration at the DT5.1 site (right) in soybean chromosome 6 (Chr6) after Cas12a released the GOI from the T‐DNA. (c) Sequence analysis of the eight single copy target events revealed RB‐LB junctions with three different re‐arrangements at the DT5.1 site. (d) Proposed T‐circle formation before integration. Left: T‐strand from T‐DNA in (b) initiated from RB and stopped at LB; middle: converted to T‐DNA, RB‐LB end joining to form T‐circle; right: DT5.1 sites in the vector were cut, re‐arrange, re‐ligate at one end and insert into the chromosomal DT5.1. (e) The T‐circle vector design to reduce random insertion events and enrich target events. (f) Summarized frequencies of transformation, SSI events, single copy events and the events with backbone. P‐At.Act7, *Arabidopsis* Actin 7 promoter; I‐Act7, P‐At.Act7 intron; *aadA*, Encoding aminoglycoside‐3″‐adenylyltransferase conferring spectinomycin resistance; *T‐nos*, Nopaline synthase terminator; Cre, *Cre* autoexcision cassette; Cas12a and gRNA, *LbCas12a* And guide RNA expression cassettes, respectively; I‐Act7 3′ and I‐Act7 5′, 270 bp of Actin 7 intron 3′ and 5′ sequence, respectively.

To improve the SSI frequency, we tested a vector design bearing two DT5.1 sites with PAMs flanking the GOI in the T‐DNA. We expected that the T‐DNA would integrate randomly in the genome and the nuclease would cut and release the GOI that could be targeted at the chromosomal DT5.1 site by NHEJ. The two unlinked insertions could then be segregated from each other in subsequent generations (Figure 1b). Eight single copy SSI events were obtained with an overall single copy SSI frequency at about 0.8%. However, detailed sequence analyses revealed that all eight SSI events had unique end joining between the RB and LB ends of the T‐DNA (Supplementary 1), suggesting that T‐circles occurred, which were further subjected to re‐linearization by cutting either or both DT5.1 sites before integration (Figure 1d).

The data from the double cut vector suggested that the single copy SSI involved an intermediate that had a RB‐LB junction. We hypothesized that such an intermediate is favourable for SSI. Thus, a vector designed to enrich for T‐circle formation may increase SSI frequency. To test this, we designed a novel vector by splitting the *aadA* selectable marker within its promoter intron (Figure S1) and placed the two parts of the split marker cassette at the RB and LB ends of the T‐DNA (Figure 1e). We reasoned that the formation of a RB‐LB junction by NHEJ would reconstitute the selectable marker expression cassette, allowing selection for such junctions. To facilitate linearization after T‐DNA circularization for genome integration, we included a single DT5.1 site with PAM into the T‐DNA (Figure 1e, vertical arrowhead). With this new design, a single T‐DNA integration in the soybean genome cannot support plant regeneration under selection, unless the T‐DNA undergoes circularization by RB‐LB end joining and re‐linearization at the vector DT5.1 site to form a functional marker gene, in which both RB and LB residue inside the intron is spliced out. Alternatively, a functional marker gene may also be reconstituted by multiple T‐DNA tandem ligation which is common in *Agrobacterium* T‐DNA integration (De Neve *et al*., 1997). Finally, a Cre autoexcision cassette together with two *loxP* sites flanking the editing accessory genes is embedded in the T‐DNA to obtain marker‐free plants in progeny (Figure 1e).

To reduce a potential adverse effect of long border residue sequences on intron splicing, two binary vectors (Figure S2) with identical elements except for the border residue length inside the intron were made: Plasmid 1 has 27 bp border residue in the intron (Figure S3), while plasmid 2 has 289 bp border residue (Figure S4). Both plasmids had lower transformation frequency (TF) than the control (Figure 1f). The TF decrease was more pronounced for plasmid 2, possibly reflecting a negative effect of a longer border residue inside the intron. Both plasmids showed doubled vector backbone frequency and 50% reduction in single copy transgenic events (Figure 1f), which likely was due to enrichment of a functional *aadA* marker gene by selecting multiple copy tandem inserts (De Neve *et al*., 1997).

Recovery of SSI events with SC‐BF‐PGE were increased 10‐fold (2.97%) using the plasmid 1, and 6‐fold (2.08%) using the plasmid 2, compared with the control of 0.3% SSI frequency (Figure 1f, Table S1). Additional SSI events (1.49% and 0.89%, respectively) were also obtained from two copy insert events: one copy of T‐DNA at the DT5.1 target site, and the other copy in other chromosome location, which would require one more generation to segregate out the transgene at the non‐target locus (Supplementary 3, S4, S5, S6). Furthermore, nine additional SSI events (2.67%) of one or two inserts with 1 to 30 bp deletion at the *loxP* site in the plasmid 1 (Table S2) were not included since they could not be expected to be substrates for marker removal by Cre recombinase.

Three types of junctions were analysed in detail at the sequence level: both insert ends and the genomic DT5.1 target site junctions, and the RB‐LB junction inside the *aadA* promoter intron (Figure S4). None of the 123 different junctions from 41 targeted events of both plasmids had a perfect DNA repair. Eleven out of 41 RB have intact RB residues while all 41 LB residues had deletions at all RB‐LB junctions (Supplementary 4, S2, S5, S8, S11), which is consistent with previous observations that the RB is more conserved (Gelvin, 2021; Singer *et al*., 2022). The end joining with PAM‐proximal ends where Cas12a still remained after cutting the target shows more nucleotide deletion than the PAM‐distal end joining, consistent with the observation from CRISPR/Cas9 NHEJ junctions (Song *et al*., 2021).

The T‐circle vector design with a split selectable marker gene has exemplified a valuable biotechnology application of the T‐circles. The reduced TF and higher frequency of targeted events may greatly reduce cost for SC‐BF‐PGE plant production by requiring less planting and analyses.

## Conflict of interest

The authors declare competing interests as employees of Bayer Crop Science. A US patent application has been submitted.

## Author contributions

X.Y. designed the experiments and drafted the manuscript. J.B. did all sequence analyses. L.G. conceived the T‐circle vector strategy design.

## Supporting information


**Appendix S1:** Materials and Methods; Supplementary 1‐4 for genetic elements, Table S1‐S6, Supplemental Figures, and detailed NHEJ junction analyses.

## Data Availability

The authors declare that the data supporting the findings are available within the paper and its Supplementary Information; or are available from the corresponding author upon reasonable request. Source data underlying all figures are available in Supplementary Data. The proprietary germplasm A3555 and the plasmids involving in the 3rd party licenses cannot be distributed. The DT5.1 target region is identical to public available germplasm Williams 82.
