# Supplementary material for: T‐circle vector strategy increases NHEJ‐mediated site‐specific integration in soybean
Source: Plant Biotechnol J. 2024 Feb 19;22(7):1926–8. doi: 10.1111/pbi.14311 (PMC11182580; doi:10.1111/pbi.14311)

Supporting information

Article type: Brief Communication

Title: T-circle vector strategy increases NHEJ-mediated site-specific integration in soybean

Authors: Xudong Ye*, John Bradley, Larry Gilbertson

Bayer Crop Science, 700 Chesterfield Parkway West, Chesterfield, MO 63017, USA

Materials and Methods

Supplementary 1: Analyses of the eight single copy site-specific integration events from the double cut vector at DT5.1 site of soybean chromosome 6

Supplementary 2: Fig. S1 to S6, Table S1 to S3 and genetic element sequences

Supplementary 3: Long-read sequencing the site-specific integration locations from T-circle vector transformation.

Supplementary 4: Junction analyses of 41 soybean site-specific integration events at DT5.1 from T-circle plasmids. Each event has two genomic insert junctions and one LB-RB junction inside the marker gene intron.

Materials and Methods

Target sequence selection.

The DT5.1 region, located near end of the soybean (*Glycine max*) chromosome 6, was selected by identifying hypomethylated regions in DNA methylation datasets in our proprietary soybean germplasm A3555, which has been disclosed in the **Supplementary 1**. The LbCas12a DT5.1 target site was selected by a proprietary gRNA finder software and is unique in the soybean genome. The target site was selected for high cutting frequency in stable transformants by Illumina sequencing of the PCR products. We observed 95% mutation frequencies at this target site across multiple experiments.

Plasmid construction.

The essential genetic element sequences including *aadA*, *Cas12a*, *gRNA* cassettes, border variants, the GOI terminator are disclosed in **Supplementary 1**. The elements were either amplified by PCR using Q5 DNA polymerase (New England Lab) from existing plasmid templates, genomic DNA or synthesized (Bio Basic Inc., Markham, Ontario, Canada) with 20 to 25 bp overlapping sequences between the assembly junction elements in primers. The expression cassettes/elements were assembled by hot fusion method as described previously (Fu *et al*., 2014). All binary vectors contain the *aadA* expression cassette as the

selectable marker which confers spectinomycin resistance in transgenic shoots (Brian *et al*. 2013). To remove the selectable marker, *LbCas12a* and gRNAs in progeny plants, a Cre recombinase autoexcision cassette (Ye *et al*., 2023) was added in the T-DNA and a pair of *loxP* sites were used to flank the marker gene and the gene editing accessory genes. An *ori pRi* binary vector backbone was used for all soybean binary vector construction which is single copy in *Agrobacterium* and produces approximately 50% single copy transgenic events in regular transformation (Ye *et al*., 2011). All plasmids were confirmed by fully sequencing.

The two plasmids designed with T-circle vector strategy are depicted in **Fig. S2**. The *aadA* marker gene is split into two pieces being placed at both T-DNA ends. The RB inner sequence after the 25 bp core sequence (**Supplementary 1**) is removed by PCR, which generates 3 bp residue after T-strand processing. Two plasmid elements are identical except for the left border length. In the plasmid 1, the inner LB sequence before 25 bp core border sequence was removed which leaves 22 bp LB residue after proper T-strand termination (**Fig. S3**). In plasmid 2, the original LB was used which leaves 285 bp inner LB sequence after T-DNA process (**Fig. S4**).

***Agrobacterium* preparation and soybean transformation.**

Agrobacterium tumefaciens AB30 strain is used for all experiments, which is derived from the nopaline strain ABI (Ye et al., 2008) by knocking out kanamycin resistance gene. The binary vectors were transformed into AB30 by electroporation and selected on LB medium with 30 mg/L gentamicin and 50 mg/L kanamycin to obtain single colonies. The vectors in *Agrobacterium* were verified by full plasmid sequencing. Soybean cultivar A3555 was used for transformation. *Agrobacterium* preparation and soybean transformation was described previously (Ye *et al*., 2008; Martinell *et al*., 2013) with minor modification using 150 mg/L spectinomycin instead of glyphosate for plant regeneration. Briefly, the mechanically excised meristem embryos from dry seeds were imbibed in the INO medium for 1 hour, inoculated with *Agrobacterium* using sonication. After five-day co-culture, the meristem embryo explants were selected on WPM media with 150 mg/L spectinomycin for 6 weeks. The green shoots with original hypocotyls were directly transferred into soil plugs for rooting. Leaf samples were collected after 2 weeks for DNA extraction. Independent primary transgenic plants that were produced in tissue culture are also called as transgenic events.

DNA extraction and transgene copy number determination.

Leaf samples were used for DNA extraction following the method previous described (Kouranov *et al*., 2022). The transgene copy number was determined by TaqMan® assay following the manufacturer's instruction (ThermoFisher Scientific, Waltham, MA USA). The primers 5'- AGCTAAGCGCGAACTGCAAT-3' (forward) and 5'- GGCTCGAAGATACCTGCAAGA-3' (reverse) amplifying the *aadA* marker gene in the soybean binary vectors, and further detected by MGB (minor grove binding) TaqMan® probe 6FAM-TGGAGAATGGCAGCGCAATGACA, were used for the *aadA* selectable marker gene copy number

assay. The *ori* pRi TaqMan® primers and detection probe as described previously (Ye *et al*., 2011) was used for all vector backbone detection.

DNA library construction for the genomic insertion fragments and analysis of target insertion by Illumina sequencing and PacBio Hi-Fi long-read sequencing.

Genomic DNA (gDNA) libraries were prepared starting with at least 20 ng of gDNA (Kouranov *et al*., 2022). Kapa HyperPlus kits (Roche) were used in a 96-well format to create individual gDNA libraries. Sizing by two-step bead purification (St. John & Quinn, 2008) of individual libraries was employed to facilitate sequencing on Illumina NovaSeq 6000 instrument. The sequence reads were assembled against the entire vector sequence and soybean genome using CLC Genomics Workbench 20.0.4 software (Qiagen). The sequences that map to the transformation vectors and genomic junction fragments were used to identify the T-DNA insertion location by BLASTn analysis using the flank sequences against the entire soybean genome.

Additional soybean transformation experiment was re-initiated with the plasmid 1 to harvest enough samples for PacBio HiFi long-read sequencing (https://www.pacb.com/revio/), which was used to verify the Illumina sequence assembly (**Supplementary 4**).

Additional references:

Fu, C., Donovan, W. P., Shikapwashya-Hasser, O., Ye, X. and Cole, R. H. (2014) Hot fusion: an efficient method to clone multiple DNA fragments as well as inverted repeats without ligase. *PLoS ONE.* **9**, e115318.

Kouranov, A., Armstrong, C., Shrawat, A., Sidorov, V., Huesgen, S., Lemke, B. *et al*. (2022) Demonstration of targeted crossovers in hybrid maize using CRISPR technology. *Commun. Biol.* **5**, 53.

Martinell, B. J., Petersen, M. W., Somers, D. A., Wan, Y., Williams, E. and Ye, X. (2013) Methods for plant transformation using spectinomycin selection. US Patent No. US8466345B2. (Washington, DC: U.S. Patent and Trademark Office). Available at: https://patents.google.com/patent/US8466345B2/en?oq=US8466345B2

St. John J. and Quinn T. W. (2008) Rapid capture of DNA targets. BioTechniques. **44**, 259-264.

Ye, X., Williams, E. J., Shen, J., Esser, J. A., Nichols, A. M., Petersen, M.W., Gilbertson L. A. (2008) Plant development inhibitory genes in binary vector backbone improve quality event efficiency in soybean transformation. *Transgenic Res.* **17**, 827–38.

Ye, X., Williams, E. J., Shen, J., Johnson, S., Lowe, B., Radke, S. *et al*. (2011) Enhanced production of single copy backbone-free transgenic plants in multiple crop species using binary vectors with a pRi replication origin in *Agrobacterium tumefaciens*. *Transgenic Res.* **20**, 773-86.

Ye, X., Vaghchhipawala, Z., Williams, E. J., Fu, C., Liu, J., Lu, F., Hall, E. L. *et al*. (2023) Cre-mediated autoexision of selectable marker genes in soybean, cotton, canola and maize transgenic plants. *Plant Cell Rep*. **42**, 45–55.

Supplementary 1

Analyses of the eight single copy site-specific integration events from the double cut vector at DT5.1 site of soybean chromosome 6

S1

Summary-Identified single insertion events with RB/LB junctions

Event No.	Insertion is consistent with:	Target
Event 1	Circularization of RB→LB fragment; cleavage at DT5.1 #2, insert @ gDNA DT5.1	DT5.1
Event 2	Circularization of RB→LB fragment; cleavage at DT5.1 #2, insert @ gDNA DT5.1; large LB deletion: lox site and DT5.1 #1 missing	DT5.1
Event 3	Circularization of RB→LB fragment; cleavage at DT5.1 #1, insert @ gDNA DT5.1	DT5.1
Event 4	Circularization of RB→LB fragment; cleavage at DT5.1 #1, insert @ gDNA DT5.1	DT5.1
Event 5	Circularization of RB→LB fragment; cleavage at DT5.1 #2, insert @ gDNA DT5.1	DT5.1
Event 6	Complex event with inverted partial repeat	DT5.1
Event 7	Complex? Identified linkages do not support a single model (possible rearrangement/inversion of gDNA flanking sequence?)	DT5.1
Event 8	Circularization of RB→LB fragment; cleavage at DT5.1 #2, insert @ gDNA DT5.1	DT5.1

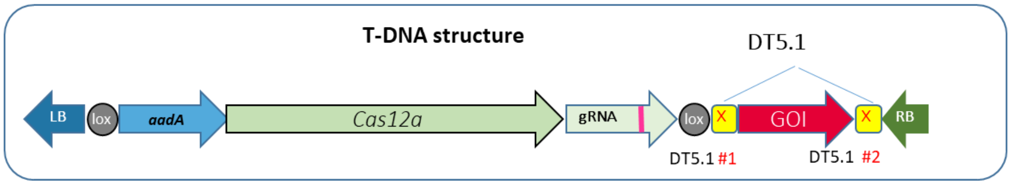

S2

Keys to Figures S3, S4, and S5:

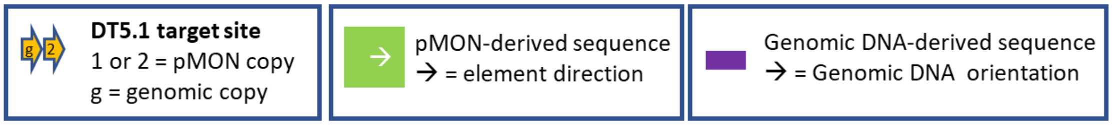

Insertions at target site in absence of evidence of a second random insertion (8 single copy targeted transgenic plants)

- Six of the eight identified events have very similar predicted insertion maps
- The similarities suggest a potential commonality of mechanism of insertion that is potentially interesting

S3

Data predict similar molecular models for four of eight transgenic plants

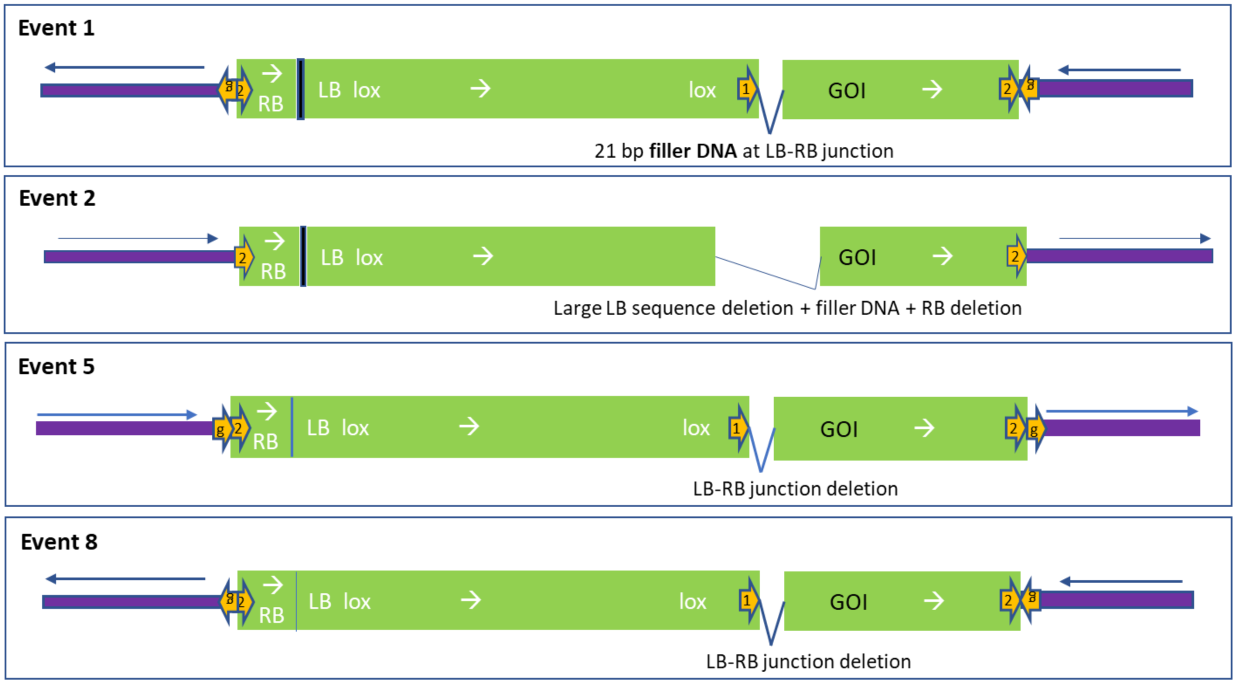

S4

Data predict similar molecular models for two of eight transgenic plants

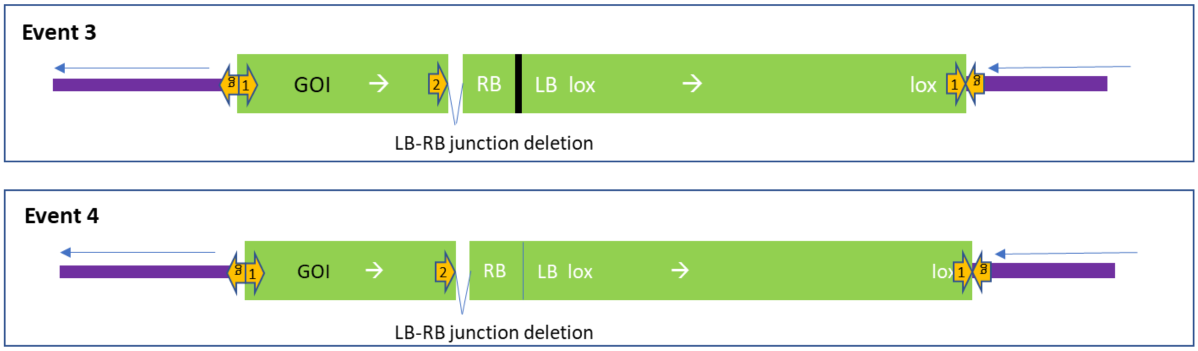

S5

Data predict complex or unassignable molecular models for two of eight transgenic plants

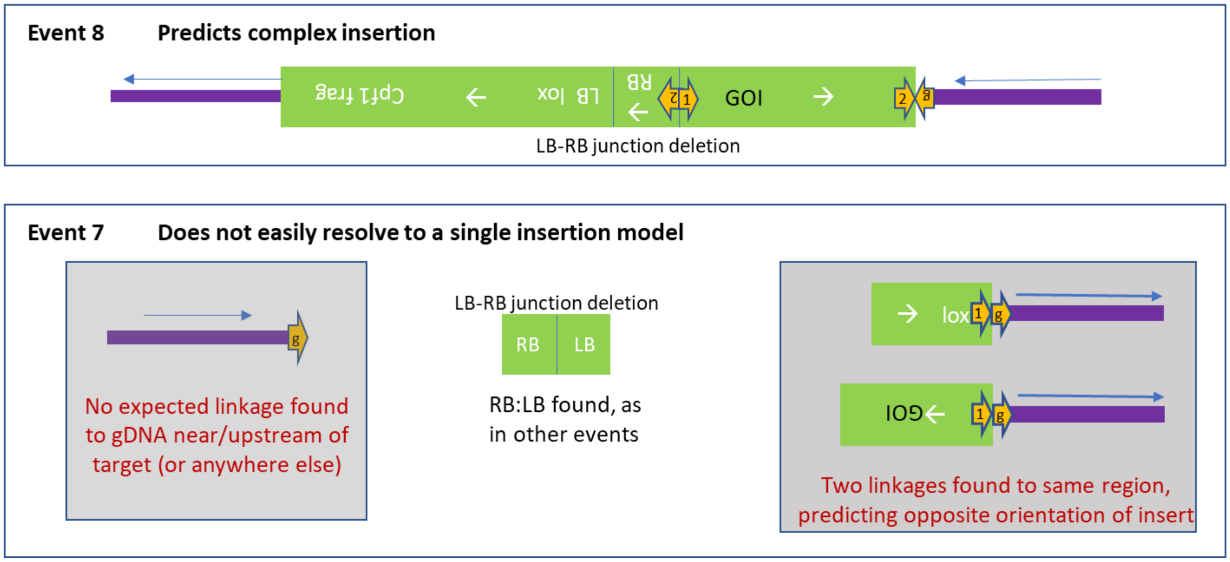

S6

Verification of RB/LB junction from 8 soybean single copy GT plants by PCR

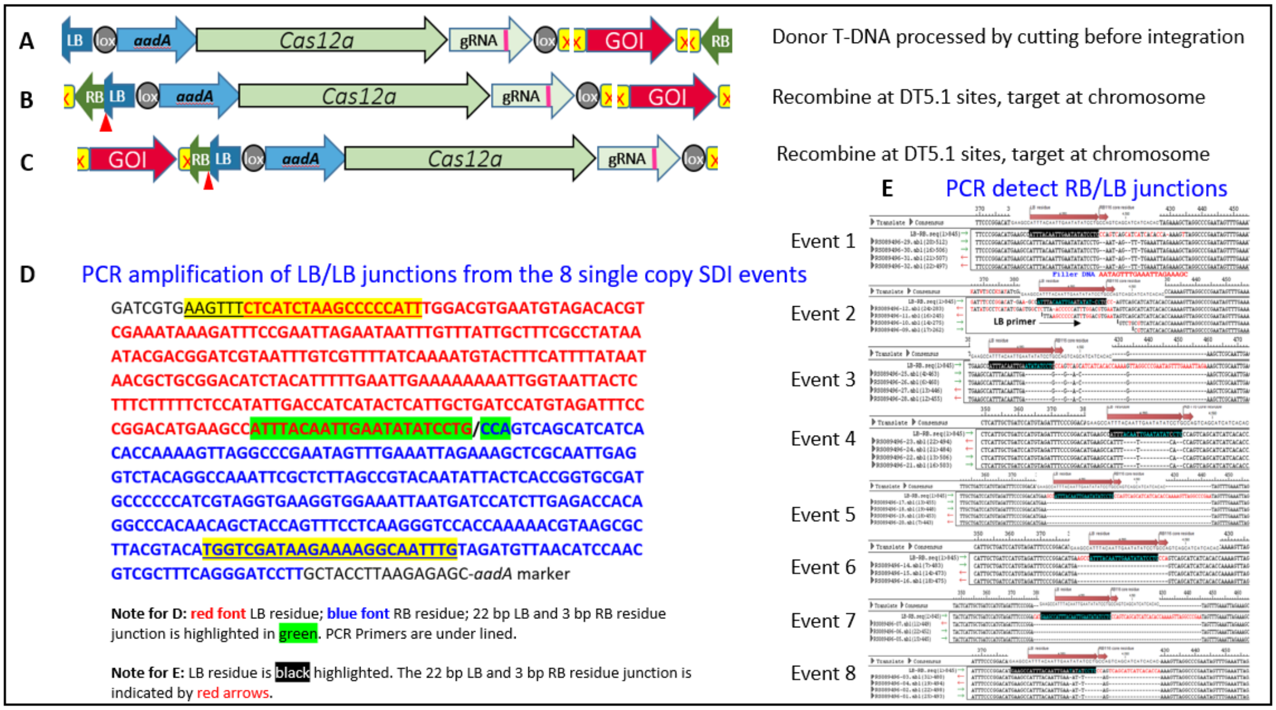

Supplementary 2

Fig. S1 to S6, Table S1 to S3 and genetic element sequences

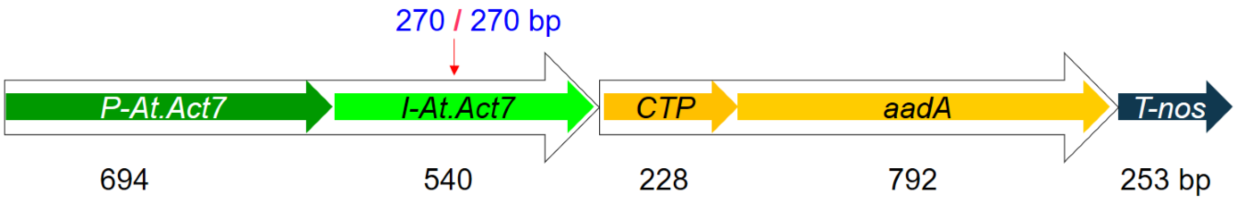

Fig. S1: The *aadA* marker gene structure. The marker gene is split into 2 parts at the middle of the *Arabidopsis* Act7 intron as indicated by a vertical arrow and used in the T-DNA of Fig. S2. ***P-At.Act7***: *Arabidopsis* actin 7 promoter; ***I-Act7* 3'**: 270 bp of actin 7 intron 3' sequence; ***I-Act7* 5'**: 270 bp of actin 7 intron 5' sequence; ***aadA***: encoding aminoglycoside-3″-adenylyltransferase for spectinomycin resistance for plant selection; ***T-nos***: nopaline synthase terminator from *Agrobacterium*;

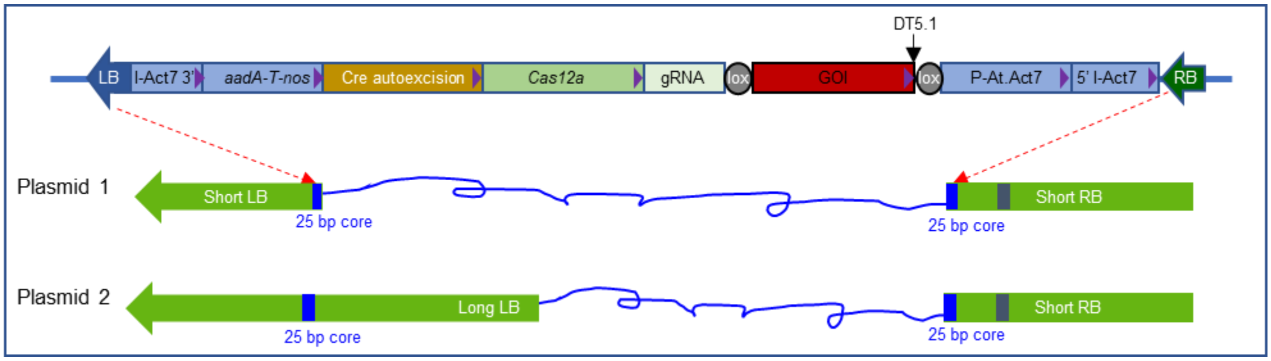

Fig. S2: T-DNA structures of the two T-circle-forming binary vectors for soybean transformation. Plasmid 1 and plasmid 2 are identical except for the left border length. The two parts of the split *aadA* marker gene are placed at T-DNA ends. The genetic elements for the *aadA* expression are indicated in Fig S1. **LB**: left border; **lox**: 34 bp *loxP* site for Cre recombinase excision; **Cre**: *Cre* recombinase driven by a developmentally regulated promoter for marker gene autoexcision; **Cas12a**: *LbCas12a* expression cassette; **gRNA**: 23 bp DT5.1 target site gRNA is expressed by a soybean U6 promoter and terminated by polyT; **GOI**: gene of interest; **RB**: right border. The border sequences and 25 bp core motif are provided in this supplementary information. The vertical arrowhead indicates the LbCas12a target site with TTTA PAM to re-linearize the circularized T-DNA after RB-LB end joining.

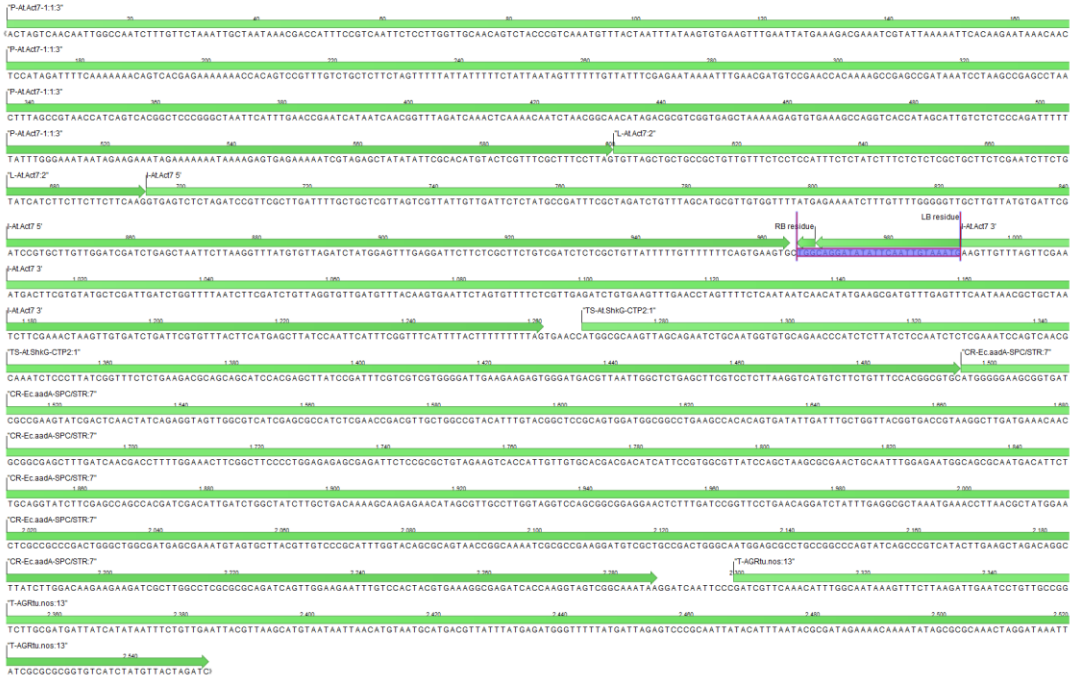

Fig. S3: The expected junction of the shortened RB and the shortened LB after T-DNA circularization and re-linearization from plasmid 1 in transgenic soybean plants. A 27 bp border residue in the middle of the intron from *Arabidopsis Act7* promoter is blue highlighted.

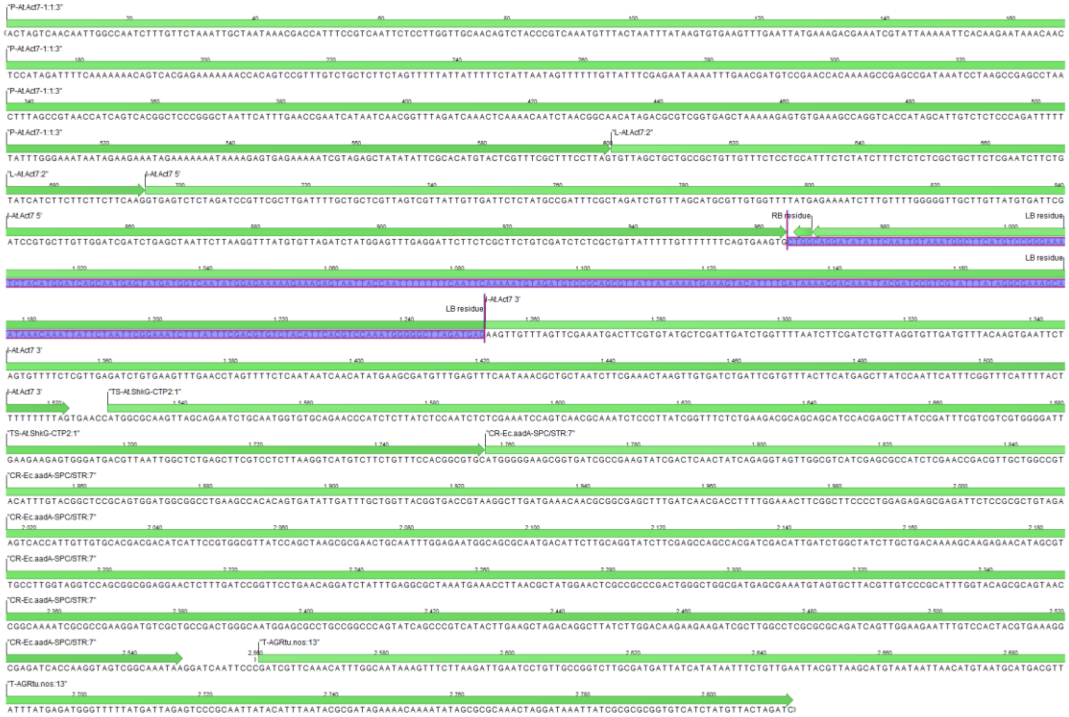

Fig. S4: The expected junction of the shortened RB and the full length of LB after T-DNA circularization and re-linearization from plasmid 2 in transgenic soybean plants. A 289 bp border residue in the middle of the intron from *Arabidopsis Act7* promoter is blue highlighted.

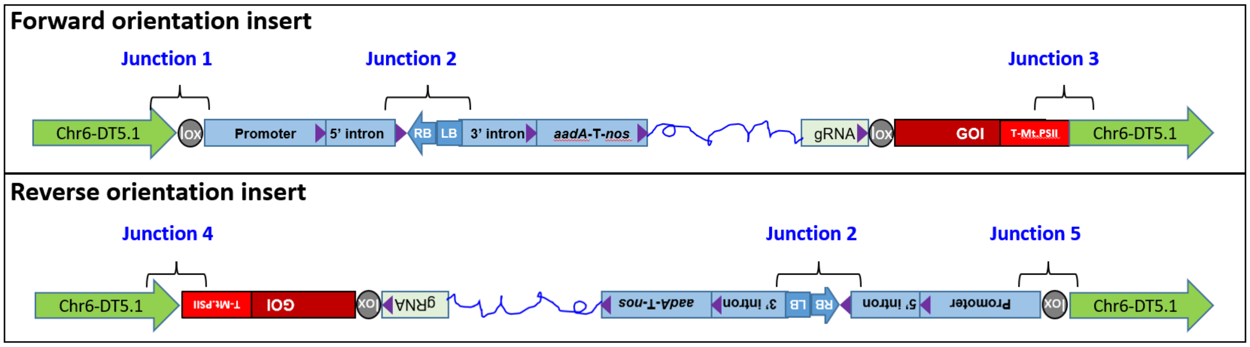

Fig. S5: Illustration of insert orientations after T-DNA circularization and re-linearization by NHEJ-mediated site-specific integration. T-Mt.PSII: the transcription terminator of *Medicago truncatula* photosystem II 5 kD protein (GenBank accession # XM_003599210.4, region 398..548); **Chr6-DT5.1**: soybean chromosome 6, DT5.1 target location. Other genetic elements have been indicated in Fig. S2.

Junction 1 **TTTA**GACTTTAGCTCCTCTGTTG/AGTC (chromosome/plasmid cut junction)
Junction 2: RB/LB junction inside the intron, see Fig. S3 and S4 for plasmid 1 and 2, respectively
Junction 3: **TTTA**GACTTTAGCTCCTCTGTTG/AGTC (plasmid cut junction/chromosome)
Junction 4: GACT/AGTC (chromosome/plasmid cut junction)
Junction 5 **TTTA**GACTTTAGCTCCTCTGTTG/CAACAGAGGAGCTAAAGTC**TAAA**
(plasmid cut/chromosome junction)

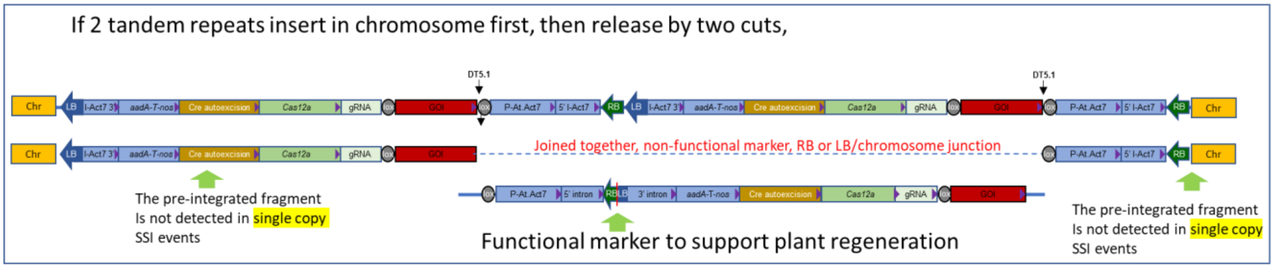

Fig. S6: Illustration of a functional marker unit may be released by double cuts from tandem repeats which may occur only in multiple copy events at rare frequency. Note: If a functional marker unit is released by simultaneous double cuts from multiple copy tandem repeats and integrated as a SSI event at the target locus, a non-functional marker fragment should be present in other chromosome with RB and LB/chromosome junctions. This may occur less likely because most single copy SSI events in our initial experiment with double cut design (**Fig. 1B, Supplementary 1**) were cut once and recombined with the rest part of inserts. The integration pattern by releasing a functional marker unit from tandem repeats with double cuts, if it occurs, is similar to the co-transformation of 2 T-DNAs: one with split marker at the T-DNA ends, and the other with the functional marker by RB-LB end-joining to form a T-circle followed by re-linearization. In all single copy SSI events that we have analyzed so far, the chromosomal junctions with RB and LB do not exist but RB-LB junction inside the *Arabidopsis Act7* intron. Therefore, it's plausible to believe that the single copy SSI events are derived from single T-DNA end-joining (T-circle) followed by re-linearization.

Table S1: Site-specific integration (SSI) frequency improvement in the T-circle vector design with split marker in soybean transformation

Construct	Initial explants	Events in soil plugs	TF	1 copy	Backbone positive	Analyzed samples	1 copy SSI rate	2 copy SSI rate
Control	30000	1824	6.10%	47%	20%	1670	0.3% (5/1670)	N/A
Plasmid 1 short LB	48000	1437	3%	23%	59%	336	2.97% (10/336)	1.49% (5/336)
Plasmid 2 long LB	48000	761	1.58%	22%	46%	336	2.08% (7/336)	0.89% (3/336)

Note: Plasmid 1 and 2 are identical except for the LB length (**Fig. S2**). TF: transformation frequency= transgenic plants/number of explants. 1 copy: frequency of single copy transgenic plants regardless of backbone. Backbone positive: frequency of *ori* pRi backbone probe detection positive plants. 1 copy SSI rate: SC-BF-PGE events. The SSI rate is defined as the SSI events/analyzed samples. 2 SSI copy events: two inserts, one in target site with perfect genetic elements, the other in other chromosome location. N/A: not available.

Table S2: Target assay summary of the events from the plasmid 1 with short LB

Sequence ID	Backbone	*aadA* copy	T-DNA location	On-target	Insert direction	Notes
#19-060	neg	2	Single, on-target	YES	REV	
#19-068	neg	1	Single, on-target	YES	REV	3 bp lox truncation
#19-069	neg	1	Single, on-target	YES	REV	
#19-070	neg	1	Single, on-target	YES	REV	13 bp insert at T-DNA::Chr junction
#19-074	neg	1	Single, on-target	YES	REV	2 bp lox truncation
#19-075	neg	1	Single, on-target	YES	REV	
#19-079	neg	1 or 2	Single, on-target	YES	REV	
#19-084	neg	1	Single, on-target	YES	REV	
#19-085	neg	3	Single, on-target	YES	REV	Duplicate T-DNA insert
#19-093	neg	1	Single, on-target	YES	REV	large deletion, rearrangement
#19-095	neg	2	Single, on-target	YES	REV	Duplicate T-DNA insert
#19-097	neg	0 or 1	Single, on-target	YES	REV	
#19-098	neg	1	Single, on-target	YES	REV	
#19-099	neg	1	Single, on-target	YES	REV	
#19-101	neg	1	Single, on-target	YES	REV	
#19-076	neg	1	Single, on-target	YES	FWD	1 bp lox truncation
#19-094	neg	1	Single, on-target	YES	FWD	9 bp lox truncation
#19-103	neg	1	Single, on-target	YES	FWD	11 bp lox truncation
#19-065	neg	2	On-target + Chr13	YES	REV	+Chr13 insert
#19-067	neg	2	On-target + Chr18 (RI)	YES	REV	2 bp lox truncation (also, RI on Chr18)
#19-072	neg	1	On-target + Chr15 (RI, short)	YES	REV	+ small insert on Chr15
#19-087	neg	2	On-target + Chr20 (RI)	YES	REV	+ insert on Chr20
#19-088	neg	2	On-target + Chr11(RI)	YES	REV	+ ambiguous insert on Chr11
#19-089	neg	1	On-target + Chr06(RI)	YES	REV	+ insert on Chr06
#19-106	neg	3	On-target + Chr12	YES	REV	+ insert on Chr12
#19-062	neg	2	On-target + Chr01 (RI)	YES	FWD	12 bp lox truncation (also, RI on Chr01)
#19-086	neg	2	On-target + Chr09 (RI)	YES	FWD	2 bp lox truncation (also, RI on Chr09)

#19-096	neg	1	On-target + Chr20(RI)	YES	FWD	30 bp lox truncation (also, RI on Chr20)
#19-057	neg	2	On-target + Chr09 + Chr19	YES	REV	too many inserts
#19-058	POS	3	On-target, BKB	YES	FWD	BKB
#19-061	POS	1	On-target + Chr12 (RI)	YES	unclear	BKB
#19-063	neg	3	On-target, large Chr gap + Chr05	YES	REV	Big Chr06 deletion
#19-064	neg	2	On-target	YES	n/a	inverted repeat?
#19-066	POS	1	On-target, BKB	YES	REV	BKB
#19-078	neg	1	On-target + Chr06 + Chr13	YES	FWD	too many inserts
#19-080	POS	2	On-target + Chr20	YES	REV	BKB
#19-081	POS	2	On-target	YES	REV	BKB
#19-083	POS	2	On-target + Chr01 (RI)	YES	REV	BKB
#19-091	neg	1	On-target + Chr08(RI) + Chr13(RI)	YES	REV	too many inserts
#19-100	POS	1	huge Chr06 deletion, BKB	YES	REV	Big Chr06 deletion, 2nd Chr06 insert
#19-102	POS	1	On-target, BKB	YES	REV	BKB
#19-059	neg	2	Chr02	no		
#19-071	neg	2	Chr06, off-target, complex	no		
#19-077	POS	1	Chr16, backbone	no		BKB
#19-082	POS	1	Chr18, Chr10 Chr16	no	.	BKB
#19-090	neg	2	Chr14	no	.	
#19-092	neg	3	Chr13, Chr07	no	.	
#19-104	POS	1	Chr10, Chr19, Chr07	no	.	
#19-105	neg	2	Chr11	no	.	

Note: Sequence ID: each represents DNA sample from independent transgenic soybean event. **Backbone**: vector backbone presence detection by Taqman®, shown as negative (neg) or positive (POS). ***aadA* copy**: *aadA* transgene copy number determined by Taqman® using *aadA* probe. **T-DNA location**: the chromosome location of T-DNA insert(s). **On-target**: T-DNA insert at the expected DT5.1 target site at the chromosome 6. **Color code**: **dark green**: single copy SC-BF-PGE SSI events; **light green**: 2 copy SSI events with one SC-BF-PGE insert; **blue highlighted**: SSI events with loxP junction deletion. **Insert direction**: two insertion orientations [forward (FWD) or reverse (REV)] as shown in Fig. S5. Other abbreviations: **Chr01**= chromosome 01, and so on; **RI**=random insert; **inv rep**=inverted repeats; BKB=backbone.

Table S3: Target assay summary of the events from the plasmid 2 with long LB

Sequence ID	backbone	*aadA* copy	T-DNA location	On-target	Insert direction	Notes
#19-004	neg	1	On-target	YES	REV	1 bp lox truncation complex
#19-006	neg	1	On-target	YES	REV	
#19-014	neg	1	On-target	YES	REV	
#19-015	neg	1	On-target	YES	REV	29 bp terminator truncation
#19-020	neg	1 or 2	On-target	YES	REV	
#19-030	neg	1	On-target	YES	REV	
#19-031	neg	1	On-target	YES	REV	weak linkages, BKB??
#19-032	neg	1	On-target	YES	REV	
#19-034	failed	failed	On-target	YES	REV	
#19-045	neg	1	On-target	YES	REV	
#19-044	neg	3	On-target + Chr01 (RI)	YES	REV	+ Chr01, other complexity?
#19-047	neg	1 or 2	On-target + Chr08 (inv rep)	YES	REV	+ Chr08, other complexity?
#19-029	neg	2	On-target, + Chr03 ins?	YES	FWD	5 bp truncation lox

#19-035	neg	1	On-target + R15 (RI)	YES	FWD	+ Chr15, other complexity?
#19-002	neg	1	complex on-target + Chr18	YES	complex	complex on target
#19-016	neg	2	on-target + Chr04+Chr20 (RI)	YES	REV	3 inserts
#19-022	neg	1	Chr06 on-target, Chr01, Chr06 (RI)	YES	REV	complex on target?
#19-025	POS	1	On-target, backbone	YES	REV	BKB
#19-026	POS	1	On-target, backbone	YES	REV	BKB
#19-027	POS	1	On-target, backbone	YES	REV	BKB
#19-040	POS	1	On-target + Chr04 (RI)	YES	REV	BKB
#19-001	neg	2	Chr15 (RI)	no		
#19-003	neg	1	Chr07, Chr06 (off-target)	no		
#19-005	neg	1	Chr20 (RI)	no		
#19-007	neg	2	Chr11 (RI)	no		
#19-008	neg	1	Chr02 (RI)	no		
#19-009	neg	0 or 1	Chr10 (RI)	no		
#19-010	neg	2	Chr20 (Inv rep)	no		
#19-011	neg	3	Chr07 (RI)	no		
#19-012	neg	2	Chr18 (inv rep, truncation)	no		
#19-013	POS	1	RI	no		
#19-017	neg	1	Chr10 (RI)	no		
#19-018	neg	1	Chr12 RI	no		
#19-019	POS	1	Chr04 RI	no		
#19-021	neg	2	Chr17 (RI)	no		
#19-023	neg	2	Chr07 (RI)	no		
#19-024	POS	1	Chr19 (RI)	no		
#19-028	POS	2	Chr19 (RI)	no		
#19-033	neg	1	Chr13 (RI)	no		
#19-036	neg	3	Chr13 (RI)	no		
#19-037	neg	3	Chr13 (RI)	no		
#19-038	neg	3	Chr18 Chr15 (inv rep)	no		
#19-039	POS	1	Chr10 (RI), BKB	no		
#19-041	neg	1	Chr17 inv rep/truncation	no		
#19-042	neg	2	Chr02 (RI)	no		
#19-043	POS	failed	Chr01 (RI), Chr05 (inv rep)	no		
#19-046	neg	3	Chr09 complex	no		
#19-048	POS	1	Chr13, Chr07, Chr09 (RI)	no		

Note: Sequence ID: each represents DNA sample from independent transgenic soybean event. **Backbone**: vector backbone presence detection by Taqman®, shown as negative (neg) or positive (POS). ***aadA* copy**: *aadA* transgene copy number determined by Taqman® using *aadA* probe. **T-DNA location**: the chromosome location of T-DNA insert(s). **On-target**: T-DNA insert at the expected DT5.1 target site at the chromosome 6. **Color code**: **dark green**: single copy SC-BF-PGE SSI events; **light green**: 2 copy SSI events with one SC-BF-PGE insert; **blue highlighted**: SSI events with loxP junction deletion. **Insert direction**: two insertion orientations [forward (FWD) or reverse (REV)] as shown in Fig. S5. Other abbreviations: **Chr01**= chromosome 01, and so on; **RI**=random insert; **inv rep**=inverted repeats; BKB=backbone.

Genetic element sequences

Full length right border (RB) sequence (25 bp core border sequence is highlighted, GenBank accession no. CP033030.1, region 99813..100317) (Holsters et al. (1983)

5′CGAAGCTCGGTCCCGTGGGTGTTCTGTCGTCTCGTTGTACAACGAAATCCATTCCCATTCCGCGCTCAAGATGGC
TTCCCCTCGGCAGTTCATCAGGGCTAAATCAATCTAGCCGACTTGTCCGGTGAAATGGGCTGCACTCCAACAGAAAC
AATCAAACAAACATACACAGCGACTTATTCACACGAGCTCAAATTACAACGGTATATATCCTG**CCA**GTCAGCATCAT
CACACCAAAAGTTAGGCCCGAATAGTTTGAAATTAGAAAGCTCGCAATTGAGGTCTACAGGCCAAATTCGCTCTTAG
CCGTACAATATTACTCACCGGTGCGATGCCCCCCATCGTAGGTGAAGGTGGAAATTAATGATCCATCTTGAGACCAC
AGGCCCACAACAGCTACCAGTTTCCTCAAGGGTCCACCAAAAACGTAAGCGCTTACGTACATGGTCGATAAGAAAAG
GCAATTTGTAGATGTTAACATCCAACGTCGCTTTCAGGGATCCTT 3′

Shortened right border (RB) sequence (25 bp core border sequence is highlighted)

5′CGAAGCTCGGTCCCGTGGGTGTTCTGTCGTCTCGTTGTACAACGAAATCCATTCCCATTCCGCGCTCAAGATGGC
TTCCCCTCGGCAGTTCATCAGGGCTAAATCAATCTAGCCGACTTGTCCGGTGAAATGGGCTGCACTCCAACAGAAAC
AATCAAACAAACATACACAGCGACTTATTCACACGAGCTCAAATTACAACGGTATATATCCTG**CCA** 3′

Full length left border (LB) sequence (25 bp core border sequence is highlighted, GenBank accession no. CP033030.1, region 86792..87233) (Holsters et al. (1983)

5′CTCATCTAAGCCCCCATTTGGACGTGAATGTAGACACGTCGAAATAAAGATTTCCGAATTAGAATAATTTGTTTA
TTGCTTTCGCCTATAAATACGACGGATCGTAATTTGTCGTTTTATCAAAATGTACTTTCATTTTATAATAACGCTGC
GGACATCTACATTTTTGAATTGAAAAAAAATTGGTAATTACTCTTTCTTTTTCTCCATATTGACCATCATACTCATT
GCTGATCCATGTAGATTTCCCGGACATGAAGCCATTTACAATTGAATATATCCTG**CCG**CCGCTGCCGCTTTGCACCC
GGTGGAGCTTGCATGTTGGTTTCTACGCAGAACTGAGCCGGTTAGGCAGATAATTTCCATTGAGAACTGAGCCATGT
GCACCTTCCCCCCAACACGGTGAGCGACGGGGCAACGGAGTGATCCACATGGGACTTTT 3′

Shortened left border (LB) sequence (25 bp core border sequence is highlighted)

5′CATTTACAATTGAATATATCCTG**CCG**CCGCTGCCGCTTTGCACCCGGTGGAGCTTGCATGTTGGTTTCTACGCAG
AACTGAGCCGGTTAGGCAGATAATTTCCATTGAGAACTGAGCCATGTGCACCTTCCCCCCAACACGGTGAGCGACGG
GGCAACGGAGTGATCCACATGGGACTTTT 3′

Reference:

Holsters, M., Villarroel, R., Gielen, J. et al. (1983) An analysis of the boundaries of the octopine TL-DNA in tumors induced by *Agrobacterium tumefaciens*. Mol Gen Genet **190**, 35–41.

The *aadA* selectable marker gene expression cassette sequence (Fig. S1, S2, S3, S4):

Arabidopsis thaliana actin 7 promoter in UPPER CASE
CTP-chloroplast transit peptide in *lower case, italic*
aadA coding sequence in **UPPER CASE, bold**
nos terminator: lower case, underlined

5′ACTAGTCAACAATTGGCCAATCTTTGTTCTAAATTGCTAATAAACGACCATTTCCGTCAATTCTCCTTGGTTGCA
ACAGTCTACCCGTCAAATGTTTACTAATTTATAAGTGTGAAGTTTGAATTATGAAAGACGAAATCGTATTAAAAATT
CACAAGAATAAACAACTCCATAGATTTTCAAAAAAACAGTCACGAGAAAAAAACCACAGTCCGTTTGTCTGCTCTTC
TAGTTTTTATTATTTTTCTATTAATAGTTTTTTGTTATTTCGAGAATAAAATTTGAACGATGTCCGAACCACAAAAG
CCGAGCCGATAAATCCTAAGCCGAGCCTAACTTTAGCCGTAACCATCAGTCACGGCTCCCGGGCTAATTCATTTGAA
CCGAATCATAATCAACGGTTTAGATCAAACTCAAAACAATCTAACGGCAACATAGACGCGTCGGTGAGCTAAAAAGA
GTGTGAAAGCCAGGTCACCATAGCATTGTCTCTCCCAGATTTTTTATTTGGGAAATAATAGAAGAAATAGAAAAAAA
TAAAAGAGTGAGAAAAATCGTAGAGCTATATATTCGCACATGTACTCGTTTCGCTTTCCTTAGTGTTAGCTGCTGCC
GCTGTTGTTTCTCCTCCATTTCTCTATCTTTCTCTCTCGCTGCTTCTCGAATCTTCTGTATCATCTTCTTCTTCTTC

AAG**GT**GAGTCTCTAGATCCGTTCGCTTGATTTTGCTGCTCGTTAGTCGTTATTGTTGATTCTCTATGCCGATTTCGC
TAGATCTGTTTAGCATGCGTTGTGGTTTTATGAGAAAATCTTTGTTTTGGGGGTTGCTTGTTATGTGATTCGATCCG
TGCTTGTTGGATCGATCTGAGCTAATTCTTAAGGTTTATGTGTTAGATCTATGGAGTTTGAGGATTCTTCTCGCTTC
TGTCGATCTCTCGCTGTTATTTTTGTTTTTTTCAGTGAAGTGAAGTTGTTTAGTTCGAAATGACTTCGTGTATGCTC
GATTGATCTGGTTTTAATCTTCGATCTGTTAGGTGTTGATGTTTACAAGTGAATTCTAGTGTTTTCTCGTTGAGATC
TGTGAAGTTTGAACCTAGTTTTCTCAATAATCAACATATGAAGCGATGTTTGAGTTTCAATAAACGCTGCTAATCTT
CGAAACTAAGTTGTGATCTGATTCGTGTTTACTTCATGAGCTTATCCAATTCATTTCGGTTTCATTTTACTTTTTTT
TT**AG**TGAACC*atggcgcaagttagcagaatctgcaatggtgtgcagaacccatctcttatctccaatctctcgaaat*
ccagtcaacgcaaatctcccttatcggtttctctgaagacgcagcagcatccacgagcttatccgatttcgtcgtcg
tggggattgaagaagagtgggatgacgttaattggctctgagcttcgtcctcttaaggtcatgtcttctgtttccac
*ggcgtgc***ATGGGGGAAGCGGTGATCGCCGAAGTATCGACTCAACTATCAGAGGTAGTTGGCGTCATCGAGCGCCATC**
TCGAACCGACGTTGCTGGCCGTACATTTGTACGGCTCCGCAGTGGATGGCGGCCTGAAGCCACACAGTGATATTGAT
TTGCTGGTTACGGTGACCGTAAGGCTTGATGAAACAACGCGGCGAGCTTTGATCAACGACCTTTTGGAAACTTCGGC
TTCCCCTGGAGAGAGCGAGATTCTCCGCGCTGTAGAAGTCACCATTGTTGTGCACGACGACATCATTCCGTGGCGTT
ATCCAGCTAAGCGCGAACTGCAATTTGGAGAATGGCAGCGCAATGACATTCTTGCAGGTATCTTCGAGCCAGCCACG
ATCGACATTGATCTGGCTATCTTGCTGACAAAAGCAAGAGAACATAGCGTTGCCTTGGTAGGTCCAGCGGCGGAGGA
ACTCTTTGATCCGGTTCCTGAACAGGATCTATTTGAGGCGCTAAATGAAACCTTAACGCTATGGAACTCGCCGCCCG
ACTGGGCTGGCGATGAGCGAAATGTAGTGCTTACGTTGTCCCGCATTTGGTACAGCGCAGTAACCGGCAAAATCGCG
CCGAAGGATGTCGCTGCCGACTGGGCAATGGAGCGCCTGCCGGCCCAGTATCAGCCCGTCATACTTGAAGCTAGACA
GGCTTATCTTGGACAAGAAGAAGATCGCTTGGCCTCGCGCGCAGATCAGTTGGAAGAATTTGTCCACTACGTGAAAG
GCGAGATCACCAAGGTAGTCGGCAAATAAGGATCAATTCCCgatcgttcaaacatttggcaataaagtttcttaaga
ttgaatcctgttgccggtcttgcgatgattatcatataatttctgttgaattacgttaagcatgtaataattaacat
gtaatgcatgacgttatttatgagatgggtttttatgattagagtcccgcaattatacatttaatacgcgatagaaa
acaaaatatagcgcgcaaactaggataaattatcgcgcgcggtgtcatctatgttactagatc 3’

LbCas12a expression cassette
Medicago truncatula ubiquitin 2 promoter *(italic);* GenBank accession # AC174341.8, region 40059..41649
The nuclear localization sequence (NLS) is highlighted)
***LbCas12a* in UPPER CASE**
T-Mt.AC145767.28 in lower case, underlined; GenBank accession # AC145767, region 93536..94035

ACTGTTAATAATTTTTAAACGTCAGCGCACTAAAAAAACGAAAAGACGGACACGTGAAAATAAAAAACACACACTAG
TTTATGACGCAATACTATTTTACTTATGATTTGGGTACATTAGACAAAACCGTGAAAGAGATGTATCAGCTATGAAA
CCTGTATACTTCAATACAGAGACGTACTCATATCGGATACGTACGCACGAAGTATCATATTAATTATTTTAATTTTT
AATAAATATTTTATCGGATACTTATGTGATACTCTACATATACACAAGGATATTTCTAAGATACTTTATAGATACGT
ATCCTAGAAAAACATGAAGAGTAAAAAAGTGAGACAATGTTGTAAAAATTCATTATAAATGTATATGATTCAATTTT
AGATATGCATCAGTATAATTGATTCTCGATGAAACACTTAAAATTATATTTCTTGTGGAAGAACGTAGCGAGAGAGG
TGATTCAGTTAGACAACATTAAATAAAATTAATGTTAAGTTCTTTTAATGATGTTTCTCTCAATATCACATCATATG
AAAATGTAATATGATTTATAAGAAAATTTTTAAAAAATTTATTTTAATAATCACATGTACTATTTTTTAAAAATTGT
ATCTTTTATAATAATACAATAATAAAGAGTAATCAGTGTTAATTTTTCTTCAAATATAAGTTTTATTATAAATCATT
GTTAACGTATCATAAGTCATTACCGTATCGTATCTTAATTTTTTTTTAAAAACCGCTAATTCACGTACCCGTATTGT
ATTGTACCCGCACCTGTATCACAATCGATCTTAGTTAGAAGAATTGTCTCGAGGCGGTGCAAGACAGCATATAATAG
ACGTGGACTCTCTTATACCAAACGTTGTCGTATCACAAAGGGTTAGGTAACAAGTCACAGTTTGTCCACGTGTCACG
TTTTAATTGGAAGAGCTGCCGTTGGCGTAATATAACAGCCAATCGATTTTTGCTATAAAAGCAAATCAGGTAAACTA
AACTTCTTCATTCTTTTCTTCCCCATCGCTACAAAACCGGTTCCTTTGGAAAAGAGATTCATTCAAACCTAGCACCC
AATTCCGTTTCAAGGTATAATCTACTTTCTATTCTTCGATTATTTTATTATTATTAGCTACTATCGTTTAATCGATC
TTTTCTTTTGATCCGTCAAATTTAAATTCAATTAGGGTTTTGTTCTTTTCTTTCATCTGATTGAAATCCTTCTGAAT
TGAACCGTTTACTTGATTTTACTGTTTATTGTATGATTTAATCCTTTGTTTTTCAAAGACAGTCTTTAGATTGTGAT

TAGGGGTTCATATAAATTTTTAGATTTGGATTTTTGTATTGTATGATTCAAAAAATACGTCCTTTAATTAGATTAGT
ACATGGATATTTTTTACCCGATTTATTGATTGTCAGGGAGAATTTGATGAGCAAGTTTTTTTGATGTCTGTTGTAAA
TTGAATTGATTATAATTGCTGATCTGCTGCTTCCAGTTTTCATAACCCATATTCTTTTAACCTTGTTGTACACACAA
*TGAAAAATTGGTGATTGATTCATTTGTTTTTCTTTGTTTTGGATTATACAGGGTaccaaaaa***ATGGCGGGATCTAAG**
AAGAGAAGAATTAAACAAGATTCGAAGCTCGAGAAGTTCACCAACTGCTACTCGCTGAGCAAGACGCTGCGGTTCAA
GGCGATCCCCGTCGGGAAGACCCAGGAGAACATCGACAACAAGCGGCTCCTGGTCGAGGACGAGAAGCGCGCCGAGG
ACTACAAGGGCGTCAAGAAGCTGCTGGACCGGTACTACCTCTCCTTCATCAACGACGTCCTGCACTCGATCAAGCTC
AAGAACCTGAACAACTACATCTCGCTGTTCCGCAAGAAGACACGGACCGAGAAGGAGAACAAGGAGCTCGAGAACCT
CGAGATCAACCTGCGCAAGGAGATCGCGAAGGCGTTCAAGGGCAACGAGGGGTACAAGAGCCTGTTCAAGAAAGACA
TCATCGAGACCATCCTGCCGGAGTTCCTGGACGACAAGGACGAGATCGCGCTGGTGAACTCGTTCAACGGGTTCACC
ACGGCCTTCACCGGGTTTTTCGACAACCGGGAGAACATGTTCAGCGAGGAGGCCAAGTCGACCAGCATCGCCTTCCG
GTGCATCAACGAGAACCTCACCCGCTACATCAGCAACATGGACATCTTCGAGAAGGTGGACGCCATCTTCGACAAGC
ACGAGGTCCAGGAGATCAAGGAAAAGATCCTGAACTCGGACTACGACGTGGAAGACTTCTTTGAGGGCGAGTTCTTC
AACTTCGTCCTCACCCAGGAGGGCATCGACGTCTACAACGCCATCATCGGCGGCTTCGTGACGGAGAGCGGCGAGAA
GATCAAGGGCCTCAACGAGTACATCAACCTCTACAACCAGAAGACTAAGCAGAAGCTCCCGAAGTTCAAGCCGCTGT
ACAAGCAAGTCCTGAGCGACCGGGAGTCCCTCTCGTTCTACGGCGAGGGCTACACGAGCGACGAGGAGGTGCTGGAG
GTGTTCCGCAACACGCTGAACAAGAACAGCGAGATCTTCAGCTCGATCAAGAAACTCGAGAAGCTGTTCAAGAACTT
CGACGAGTACAGCAGCGCCGGCATCTTCGTCAAGAACGGGCCCGCGATCAGCACCATCAGCAAGGACATCTTCGGGG
AGTGGAACGTGATCCGCGACAAGTGGAACGCCGAGTACGACGACATCCACCTCAAGAAAAAGGCGGTGGTCACGGAG
AAGTACGAGGACGACCGCCGGAAGTCCTTCAAGAAAATCGGGAGCTTCAGCCTCGAGCAGCTCCAGGAGTACGCGGA
CGCCGACCTGAGCGTGGTGGAGAAGCTCAAGGAGATCATCATCCAGAAGGTCGACGAGATCTACAAGGTCTACGGCT
CGAGCGAGAAGCTGTTCGACGCGGACTTCGTGCTGGAGAAGTCCCTCAAGAAGAACGACGCCGTGGTGGCCATCATG
AAGGATCTGCTCGACAGCGTGAAGTCGTTCGAGAACTACATCAAGGCATTCTTTGGGGAGGGCAAGGAGACGAACCG
GGACGAGTCCTTCTACGGGGACTTCGTGCTCGCGTACGACATCCTCCTGAAGGTCGACCACATCTACGACGCGATCC
GGAACTACGTCACGCAGAAGCCCTACAGCAAGGACAAGTTCAAGCTCTACTTCCAGAACCCGCAGTTCATGGGCGGG
TGGGACAAGGACAAGGAGACCGACTACCGGGCCACGATCCTGCGGTACGGGTCCAAGTACTACCTCGCCATCATGGA
CAAGAAGTACGCCAAGTGCCTCCAGAAGATTGACAAGGACGACGTGAACGGGAACTACGAGAAGATCAACTACAAGC
TCCTCCCGGGGCCCAACAAGATGCTGCCGAAGGTGTTCTTCAGCAAGAAGTGGATGGCCTACTACAACCCCTCGGAG
GACATCCAGAAGATATACAAGAACGGCACGTTCAAAAAGGGGGACATGTTCAACCTGAACGACTGCCACAAGCTGAT
CGACTTTTTCAAGGACAGCATCAGCCGCTACCCGAAGTGGTCGAACGCCTACGACTTCAACTTCTCGGAGACGGAGA
AGTACAAGGACATTGCGGGCTTCTACCGGGAGGTGGAGGAGCAGGGCTACAAGGTCTCCTTCGAGAGCGCCTCCAAG
AAAGAGGTGGACAAGCTCGTGGAGGAGGGCAAGCTGTACATGTTCCAGATCTACAACAAGGACTTCTCGGACAAGTC
GCACGGCACCCCGAACCTCCACACGATGTACTTCAAGCTGCTGTTCGACGAGAACAACCACGGGCAGATCCGCCTCA
GCGGCGGGGCGGAGCTGTTCATGCGCCGCGCGTCCCTCAAGAAGGAGGAGCTGGTCGTGCACCCCGCCAACTCCCCG
ATCGCGAACAAGAACCCCGACAACCCCAAGAAGACAACCACCCTCTCGTACGACGTCTACAAGGACAAGCGGTTCTC
GGAGGACCAGTACGAGCTGCACATCCCGATCGCCATCAACAAGTGCCCCAAGAACATCTTCAAGATCAACACCGAGG
TGCGGGTGCTGCTCAAGCACGACGACAACCCCTACGTCATCGGGATCGACCGCGGCGAGCGGAACCTGCTCTACATC
GTGGTCGTGGACGGGAAGGGGAACATCGTGGAGCAGTACAGCCTGAACGAGATCATCAACAACTTCAACGGCATCCG
CATCAAGACGGACTACCACAGCCTCCTGGACAAGAAGGAGAAGGAGCGGTTCGAGGCGCGGCAGAACTGGACCTCCA
TCGAGAACATCAAGGAGCTGAAGGCCGGCTACATCAGCCAGGTCGTGCACAAGATCTGCGAGCTCGTGGAGAAGTAC
GACGCGGTGATCGCGCTGGAGGACTTGAACAGCGGGTTCAAGAACTCCCGGGTCAAGGTCGAGAAGCAGGTCTACCA
GAAGTTCGAGAAGATGCTGATCGACAAGCTCAACTACATGGTGGACAAGAAGTCCAACCCCTGCGCCACCGGCGGCG
CCCTCAAGGGCTACCAGATCACCAACAAGTTCGAGTCCTTCAAGTCGATGTCTACGCAGAACGGGTTCATTTTCTAC
ATCCCGGCGTGGCTCACCAGCAAGATCGACCCGAGCACGGGCTTCGTCAACCTCCTGAAGACCAAGTACACCAGCAT
CGCGGACAGCAAGAAGTTCATCTCCTCGTTCGACCGCATCATGTACGTCCCCGAGGAAGACCTGTTCGAGTTCGCCC
TCGACTACAAGAACTTCTCCCGGACGGACGCCGACTACATCAAAAAGTGGAAGCTCTACAGCTACGGCAACCGGATC
CGCATCTTCCGCAACCCCAAGAAGAACAATGTGTTCGACTGGGAGGAGGTGTGCCTGACGAGCGCCTACAAGGAGCT
CTTCAACAAGTACGGCATCAACTACCAGCAAGGGGACATCCGCGCGCTGCTCTGCGAGCAGTCCGACAAGGCGTTCT
ACTCGTCGTTCATGGCCCTGATGAGCCTCATGCTCCAGATGCGCAACAGCATCACCGGCCGGACGGACGTGGACTTC

CTGATCAGCCCGGTCAAGAACAGCGACGGCATTTTCTACGACAGCCGGAACTACGAGGCCCAGGAGAACGCCATCCT
CCCCAAGAACGCCGACGCGAACGGCGCCTACAACATCGCGCGGAAGGTGCTGTGGGCCATCGGCCAGTTTAAAAAGG
CGGAGGACGAGAAGCTGGACAAGGTCAAGATCGCCATCAGCAACAAGGAGTGGCTCGAGTACGCGCAGACGAGCGTG
AAGCACGGATCTAAGAAGAGAAGAATTAAACAAGAT**TGA**TTAAttaatcatctgaaactgttcaccatgcatgcaat
cttgtgaaatatatggttttaattagacttcaatcttatgttggctattgtactaataaaagcatgtcatgttattt
tcatttgattttatctgtactttggtttgtttgaagaataaagatgagcttgctatgcatgcatgcatgccatcgat
tatcagggtttcctttttttctttttctggcttcccatcaatttggtgtgaattagtgtgtgtgatatattatattatg
ctatttatgaaataaattgttggttatatttgatctacaatctacatacatgtgattttttatcaacaaaatatctcg
ggaaacaataccttttttggtagcaaaattcaaataatactattttaaataaatcaaagttaaccaataccttattca
agttggaggggtctcaaacaagcaaaagaattcaagttgttaatgaacttcggttaatgataaaagaattcgcattt
aaaa

Soybean U6 promoter+DT5.1 gRNA cassette (**DT5.1 target site** in bold, CRSPR RNA is highlighted)**:**
CGATAAAAATGTTTTAAACGATATATATTATAAAAAAAAAACGTTTCAAAAATAAATACAAAAATGTTTTTAAATATA
TATAATTTAACTCATTAAAGAAAATAAAAATGCAAGTGCGGTGACAAGACAAGCTAAAAGTTGCAAAAGAAATGGCA
GGGCTATAAGGCTCACCTACTCCTGGATTTACCAAATTTTGGTTCGTCCCTATACTCGAAAAATAAAACAAAATAAA
TTTCAGTATCTTCGTTTTTGTATGCTTTGACTGTGAGGCGAGGCCAACTTTCTTCTTCTGTCTGAGATGAATTTTGT
TTGCCTCCTGTGAAGGATGTATCATTCAAAGTGAATGTTTTGCAACTGCCAGTAGTCCCACATCGACCAAATATTCT
TATTACAGTGTGTTTATATAGCACCTGGAGAAGGAATGGGTTgtccTAATTTCTACTAAGTGTAGAT**GACTTTAGCT**
CCTCTGTTGAGTCTAATTTCTACTAAGTGTAGATTTTTTTT

Soybean DT5.1 target sequence (GenBank accession no. NC_038242.2, region 47935988..47938053): The **DT5.1** Cas12A target site (reversed orientation) is marked in bold, the PAM is highlighted.
TCTTATTATCTTTAATGGAATTACAATTTTTTCCATATCTAAGATCTTGATTAAAGATTTTAAGTTCAATCTCAAAC
TAATGGCATGACACTTAACTTAATCAAGTGTAATAAGGTTAACCTACACATTCTGCAGAAGATGTGCACTGCGTGAT
TTTGTTTGTTTTTACATAACATGAATATGGTCCACCTTTTTGTATGACAAATGCAAAGACAATGGTAATAGATGCAC
CGTAGGGCCATGAAGCCAATCAATCTCAGAACTGTCCGCAATTTTCTCGACTAGTGAATATATAGAGACATTGCATC
CACTATATACTTCATTGCTTACAAAATATGCATGGAAAGCTTTCCTAGCTATATTTTTGTCACAATATTATGATGAG
AACAAATTAACGAAAAGAAGTAGTCACACATGTCAAAAAGAAGCGAAGAAAAGTTAAAAATCCATTAGGGGCCAAGT
GTATTTAAGAGCTCTATTAAGCATTCCAAATTTCTTTTGACAAATTAATCAAGCAAGAAAATAAATAAATAAACATC
AATTCTCATATTATAAATTTAAGTTATTGTGCTTCAAAACTATTTAATCATATATCGTTCTCATTTTTTGACACACG
TGTTATGAATTATGAGTTTCTTTCTTTAAGATATGCTTTTATGAAGAGTTTTGATGGGAAAGGAAAAACAAGATAAA
TTTTTCGTTTTTAATAAATACTTAAATTTCTTCAATCAATATTAATAAATAAAATATTTAAAATTCATTACTGTTTT
TTAATTGAAAAACTTCAAACATTTATATCTTTATACTTTACGTTATATTTTTTTATTACTAGTAGAATAGTTTTCCT
TTTAAATATTTATAATTTATTTTTATTGCCTAAAAATTATTTATTTTTTATGTTTTACATATGTGTAAAAGATCATC
CAATGAGCAAGATACTTACAAGTTACAAGCTCGTGTTTAACTCAAAATGCATGTATTGAACTTTTATTATTATTATT
ATTTCTTTCCTTCGTTCTGTCTTTA**GACTTTAGCTCCTCTGTTGAGTC**TAAACAACACTACATCTGACCTAAGAATG
AGCCCCACCCATGAGCATTGTTTATGACAAACCCTAATAAATACATTAACTGATTAGATAATACAATGTTAGTACAT
ACAGAGAAACGTAGCCATCATGTAATAAATTTGTGTCTTCATTATCGAATGAGAGTATTTTTAGAAATATTCTCATT
TTCATTAAGTCACATAACTTCTCTTAAATTAAATAATTAGTTTTATTTTATTTATAATTTTCATTTTCTTTTCAAAT
GTTACCTCTTAAATTTATTTTCATGCATGCAAACGATGCGCATTTAATCATCAGTTATTGAGAACTCAGAAATTGCA
AACCTCTCTATAAAAAAAAATTGCAAACCTATGATCAATAATTTAAGAGTTTAACTTTTATTCATATTTTGACGGTA
AAAAGAAAATCCTGTTGTTCATATTTAAAATGATTATTACAAAAACCAACATTTGTCATATATAGTATGACATGATA
ATTTATAAGGCTTTTTGTTTACACTAAAGATATTCCCCAACATTTCTCATGCATGTTTAGATTACCCTTTGGTATCC
TTCAACTTACTTTTAGATCAACTCATATTATACCAAAGTCTGATGATAAAAAATATTATACCAAACTCAAGTTTATA
ATATACTTTTTTAATTCAAACTTACATTTACAAATTTTAATCTAAACATATACTATGTCAGATTTTATTGTTGATAA
TTTATAATTAGGTAATAAATAATAATGTAAAAGAATTACAATGTGAATGCATTCTAATTAAATTTATATTTCAAATT
TTACAACTAAATTTTCTATTTATAATTTTACTATAAAAACGTTTTAAACAGATGAAAATTTTTTATTGCAAAAGAAG
ACGACGATCCTAACATTTGTCAACAAAGTCCCTTTTAAAGTTCACACTGAGCAGGTGTGAGGGTGAGAACAGGGCTC
TACTGCTAACTATATCGAACGGCGGCAGAATCTCGCATTTAAAACATACATACTCATCAAATCA

Supplementary 3: Long-read sequencing the site-specific integration locations from T-circle vector transformation.

We analyzed three SSI events with PacBio Hi-Fi sequencing for long-reads to verify Illumina sequence assembly.

The plasmid 1 was re-transformed into soybean meristem explants as described in Materials and Method. Approximately 5000 soybean explants were used for transformation. One hundred eighty-four leaf samples were directly harvested from tissue culture containers, extracted for genomic DNA and analyzed for the DT5.1 targeted integration with Illumina sequence assembly. In total, 49 SSI events (26.6%) were identified, of which 10 (5.4%) were single copy SSI events with different DT5.1 target site junctions.

Selected events were transferred into soil plugs in a growth chamber to harvest enough leaf tissue for PacBio DNA extraction. Approximately one gram of leaf tissue was pulverized in liquid nitrogen and extracted using a CTAB-based protocol (Aboul-Maaty & Oraby, 2019) with an additional step of phenol extraction before precipitation. The genomic DNA was rinsed with 70% ethanol, air-dried and resuspended in water.

The extracted DNA samples were prepared for PacBio Hi-Fi sequencing libraries using SMRTbell prep kit 3.0 according to manufacturer instruction and loaded into PacBio REVIO system for long-read sequencing (https://www.pacb.com/revio/). The Hi-Fi reads with approximately 19 kb in length on average of the genomic data were aligned to the two directional reference sequences of the expected DT5.1 target integration (**Supplementary 2, Fig. S5**) across the 14 kb target regions from the T-circle re-linearization using CLC Genomics Workbench (https://www.Qiagen.com).

Three events were verified by the PacBio long-read sequencing as expected with the Illumina sequencing assembly. The event #9 and #33 are **single copy SSI** at the DT5.1 location, while the event #83 has **two copy insertions** with one SSI at the DT5.1 location and the other random integration in chromosome 17. Two long-reads of the event #9, a single long-read of the event #33 and a single long-read of the event #83 covered the entire 14 kb target region at the chromosome 6 DT5.1 target location (**Supplementary 3, S1**). The detailed chromosome/linearized T-circle junctions and the LB/RB junction inside the *Arabidopsis actin 7* intron have been revealed in **Supplementary 3, S2, S3 and S4, respectively**. All junctions have deletions which are consistent with previous observations using Illumina sequence assembly.

We also analyzed the co-transformed T-DNA copy at the chromosome 17 location of the event #83 (**Supplementary 3, S4, S5, S6)**. Both RB and LB junction have deletions. An 89 bp reversed sequence derived from the adjacent flank of the chromosome 17 is re-inserted between the RB junctions.

References:

Aboul-Maaty, N.A.F., Oraby, H.A.S. (2019) Extraction of high-quality genomic DNA from different plant orders applying a modified CTAB-based method. Bull Natl Res Cent 43, 25. https://doi.org/10.1186/s42269-019-0066-1

Acknowledgements

We are grateful to Drs. Ashok Shrawat and Kevin Lutke for assistance in soybean transformation, Dr. Zijin Du for PacBio Hi-Fi library preparation and sequencing, Dr. Linda Rymarquis for Illumina sequence analyses of all target events and PacBio sequence alignments, and Drs. Jenn To, Ericka Havecker and Xuefeng Zhou for supporting this supplementary work.

S1

Mapping long-reads to DT5.1 target reference sequences at the chromosome 6 location

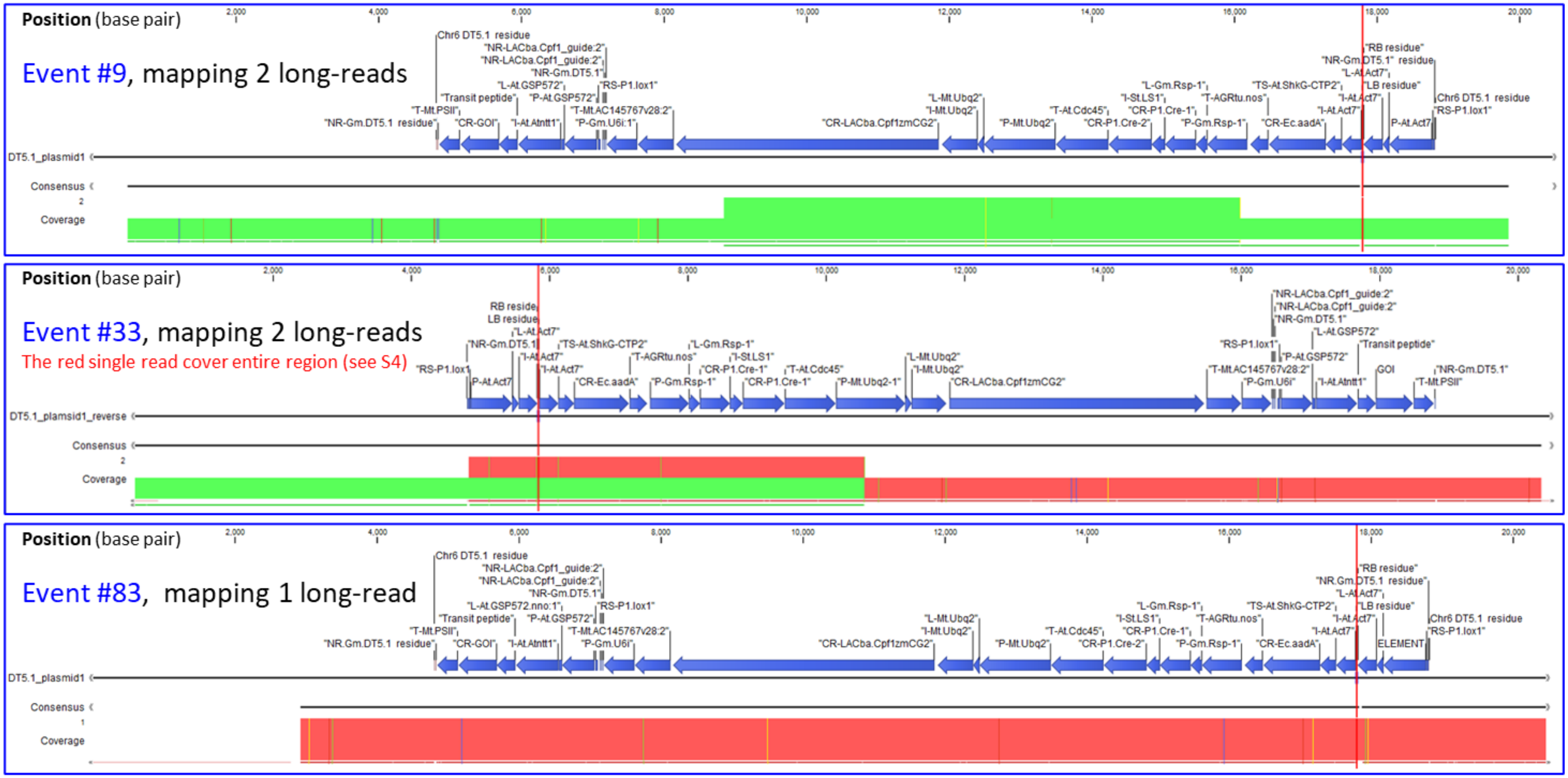

Note: LB/RB junction is indicated by a long vertical red line. Genetic elements are indicated in **Supplementary 2**. The vertical lines interrupting consensus sequences are SNPs or deletions.

S2

SSI event #9: DT5.1 target insert junction structures from PacBio Hi-Fi long-read sequencing

Two overlapped long-read mapping

Chromosome 6 and T-DNA DT5.1 cut site residue junction

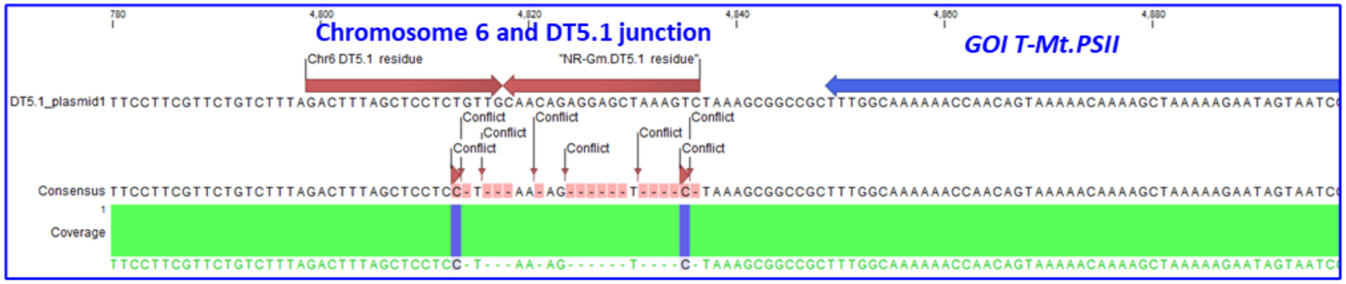

T-DNA left and right border residue junction inside the *Arabidopsis Act7* intron

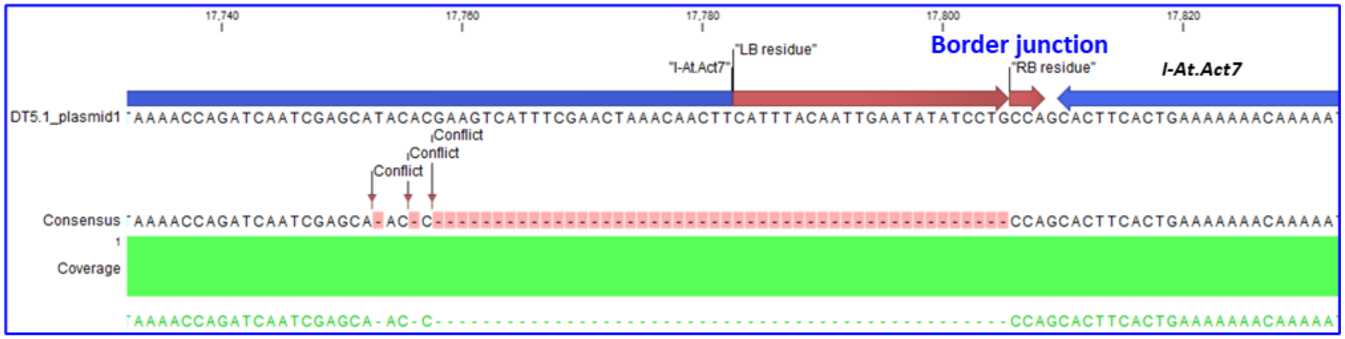

T-DNA and chromosome 6 DT5.1 cut site residue junction

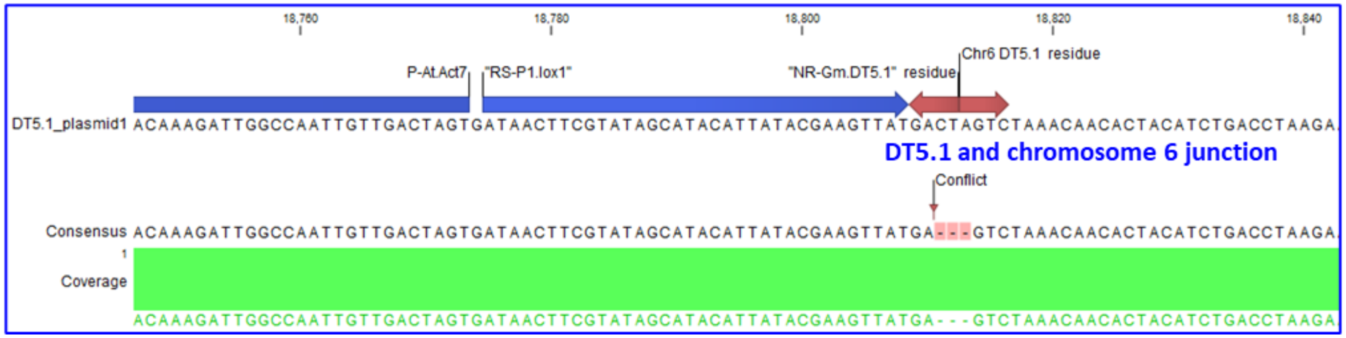

S3

SSI event #33 (reverse insert): DT5.1 target insert junction structures from PacBio Hi-Fi long-read sequencing

Chromosome 6 and T-DNA DT5.1 cut site residue junction

Note: The red single long-read has covered the entire 14 kb target region (see S1). Its 5' end is not long enough to be aligned properly by the software. Adding some protection sequences between the DT5.1 cut site and the *loxP* site inside the T-DNA could have avoided the chewback in the *loxP* site.

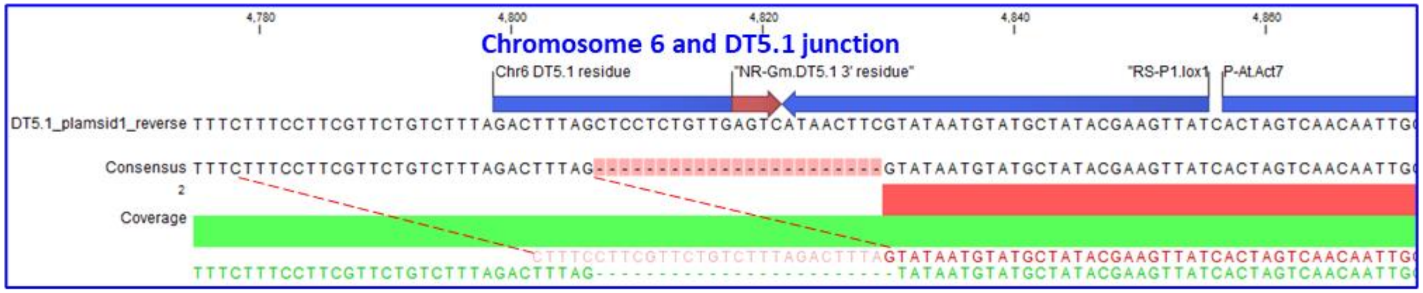

T-DNA left and right border residue junction inside the *Arabidopsis Act7* intron

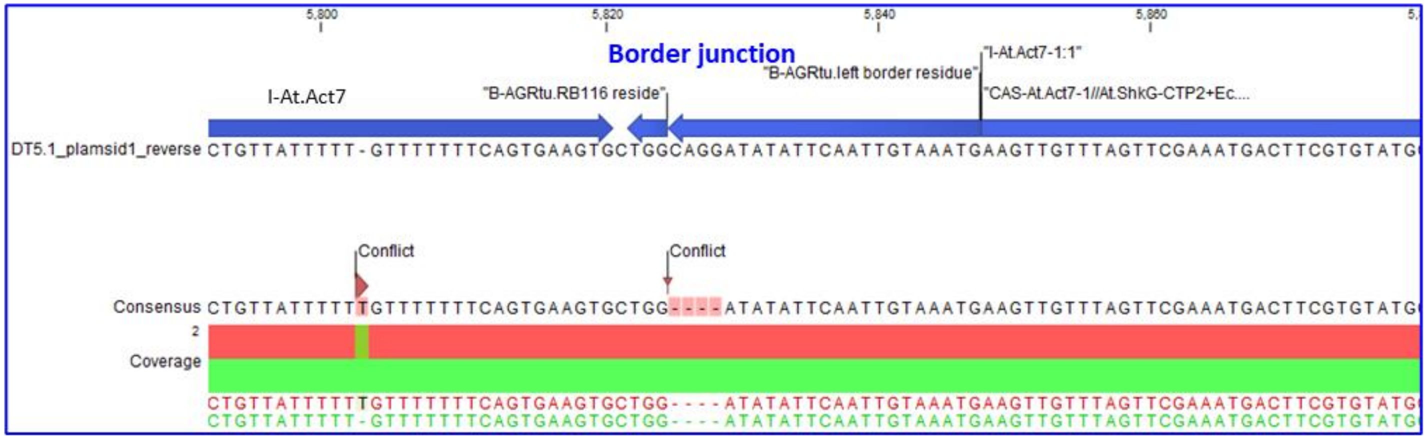

T-DNA and chromosome 6 DT5.1 cut site residue junction

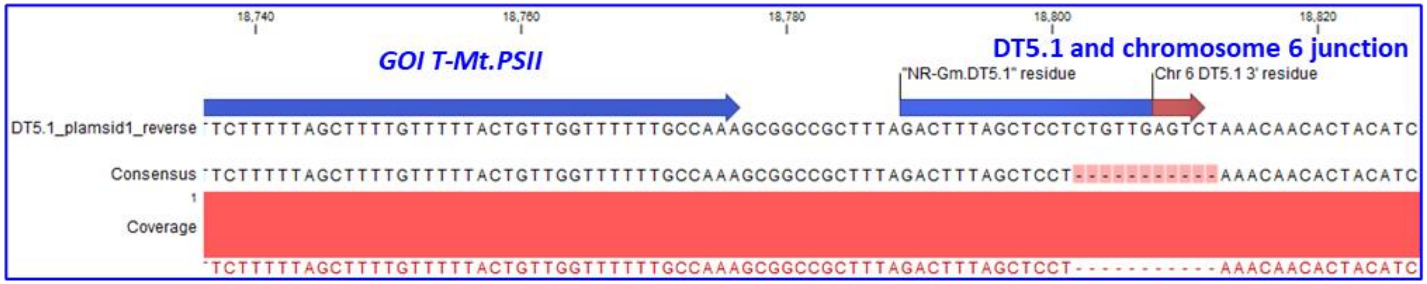

S4

SSI event #83: DT5.1 target insert junction structures from PacBio Hi-Fi long-read sequencing

The #83 plant is a two-copy event: one copy T-DNA is likely derived from a T-circle, re-linearized and site-specifically integrated at the Chr6 DT5.1 site, although we could not completely rule out the possibility of releasing the target sequence from a 2-copy tandem integration at the chromosome 17 by simultaneously cutting the DT5.1 sites inside the tandem T-DNA which occurred at rare frequency (see **Fig.1B**, **Supplementary 1** and **Supplementary 2**, **Fig. S6**); the other randomly integrated at the Chr17 without forming T-circle (see **S5** and **S6**). The SSI event at DT5.1 location can be segregated from the Chr17 insertion in T1 generation. The marker-free GOI seeds may be obtained by Cre/*loxP* autoexcision (Ye *et al.*, 2023).

Single long-read mapping

Chromosome 6 and T-DNA DT5.1 cut site residue junction

T-DNA left and right border residue junction inside the *Arabidopsis Act7* intron

T-DNA and chromosome 6 DT5.1 cut site residue junction

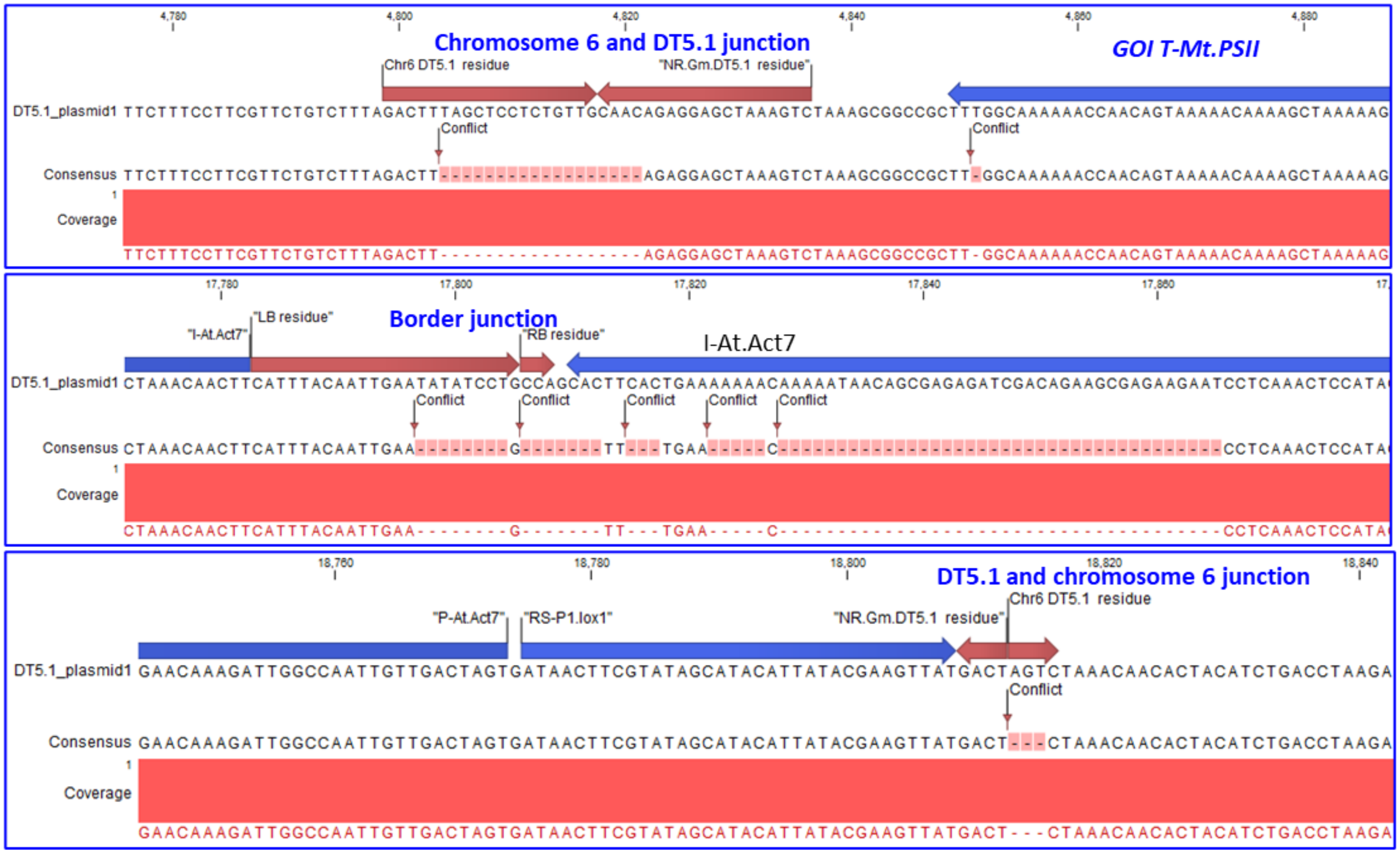

S5

Characterization of the co-transformed T-DNA copy in chromosome 17 of the SSI event # 83

The soybean transgenic plant #83 is a two-copy transgenic event: one copy SSI at the chromosome 6 DT5.1 site (see **S4**), the other integrated at soybean phospholipase A1-I beta2 mRNA 5' leader sequence (LOC100819591, GenBank # XM_003549891). This region sequence is identical between soybean cultivar Williams 82 and A3555. The three junctions are detailed in **S6**.

The structure of the linear T-DNA integration in Chr 17

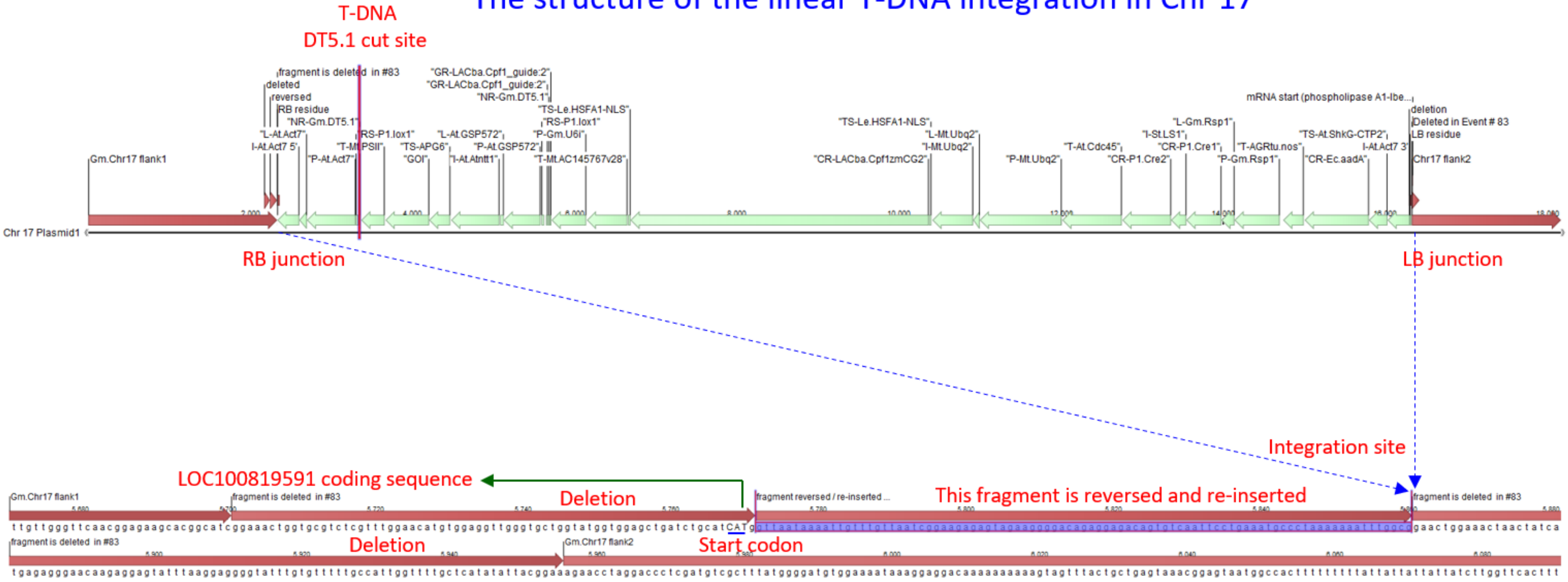

S6

Characterization of the co-transformed T-DNA copy in chromosome 17 of the SSI event # 83 (continued)

The detailed insertion junctions at *Glycine max* chromosome 17, GenBank accession # CP126442

RB junction

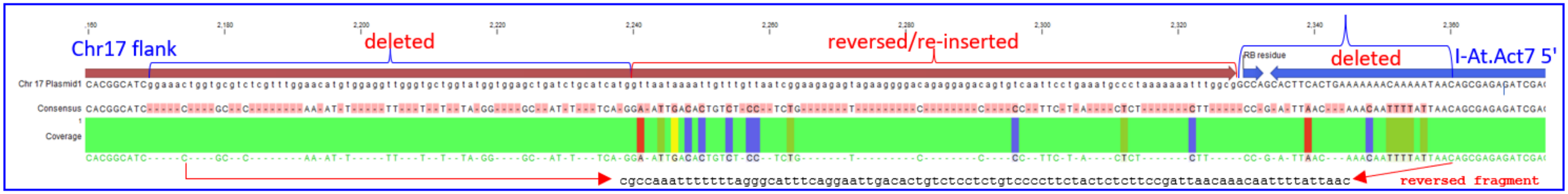

T-DNA DT5.1 junction: the INDEL abolished the DT5.1 and *loxP* sites

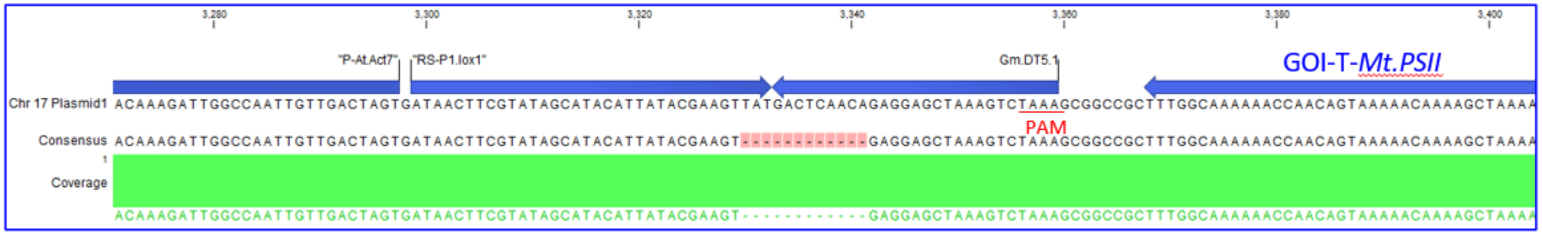

LB junction: 95 bp chr17 flank deletion

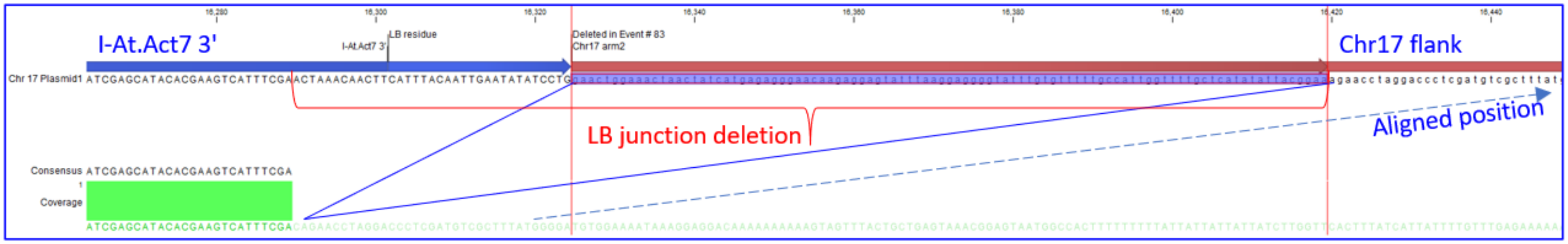

Note for the T-DNA in soybean chromosome 17: The linear T-DNA is randomly inserted into the soybean phospholipase A1-I beta2 mRNA 5' leader sequence. The RB junction has 71 bp genomic flank deletion, followed by 89 bp genomic flank reversion and further 32 bp T-DNA RB terminus chewback. The LB junction has 35 bp chewback in the T-DNA LB terminus and 95 bp deletion in the genomic flank. The T-DNA DT5.1 site is cut and imperfectly repaired with 9 bp deletion at the cut site which prevents further re-cutting by Cas12a nuclease.

Supplementary 4

Junction analyses of 41 soybean site-specific integration events at DT5.1 from T-circle plasmids. Each event has two genomic insert junctions and one LB-RB junction inside the marker gene intron.

Summary

Eleven out of 41 RB have intact RB residues while 41 LB residues had deletions at the RB-LB junctions from all 41 events (**S2, S5, S8, S11**), which is consistent with previous observations that RB is more conserved (Gelvin, 2021; Singer *et al*., 2022). The 4 bp DT5.1 PAM-distal end back-to-back junctions had deletions with an average of 7.5 bp from 32 events (**S1, S7**), while the 19 bp DT5.1 PAM-proximal end back-to-back junctions had an average of 24 bp deletion from the same events (**3, S3, S9**). Sixteen junctions expecting to reconstitute the DT5.1 site from the forward orientation inserts of 8 events (**Fig. S4**) had an average of 31 bp deletions (**S4, S6, S10, S12**), indicating that a simple directional ligation between the two Cpf1 5' overhangs did not occur (Zetsche *et al*., 2015). The largest deletions up to 339 bp are observed in the plasmid 2 with long border residues inside the *aadA* promoter intron (**S8, S11**), which is concordant with a further TF drop using the plasmid 2 and suggests that long residues inside the intron interfere the marker gene expression. The RB-LB junction from the plasmid 1 contained filler DNA of 7-37 bp at the gaps in 6 of total 27 events, which is common in T-DNA integration (Gelvin, 2021; Singer *et al*., 2022).

S1) Target events from the Plasmid 1 with short LB, reverse orientation insertions, chromosome linkages at *lox end* of T-DNA

INSERT #	Sample #	<-- Chr06	<- DT5.1	DT5.1 -> (T-DNA)	<-- P1.lox1:1 (T-DNA)	P-At.Act7-> (T-DNA)
		ATTCTTAGGTCAGATGTAGTGTTGTTTA	GACT	AGTC	ATAACTTCGTATAATGTATGCTATACGAAGTTAT	CACTAGTCAA
1	#19-060	ATTCTTAGGTCAGATGTAGTGTT.....	....	.GTC	ATAACTTCGTATAATGTATGCTATACGAAGTTAT	CACTAGTCAA
1	#19-068	ATTCTTAGGTCAGATGTAGTGTTGTTT.	....	....	...ACTTCGTATAATGTATGCTATACGAAGTTAT	CACTAGTCAA
1	#19-069	ATTCTTAG....................	....	.GTC	ATAACTTCGTATAATGTATGCTATACGAAGTTAT	CACTAGTCAA
1	#19-070	ATTCTTAGGTCAGATGTAGTGTTGTTTA	G...	AGTC	ATAACTTCGTATAATGTATGCTATACGAAGTTAT	CACTAGTCAA
1	#19-073	ATTCTTAGGTCAGATGTAGTGTTGTTTA	....	....	ATAACTTCGTATAATGTATGCTATACGAAGTTAT	CACTAGTCA
1	#19-074	ATTCTTAGGTCAGATGTAGTGTTG....	....	....	..AACTTCGTATAATGTATGCTATACGAAGTTAT	CACTAGTCAA
1	#19-075	ATTCTTAGGTCAGATGTAGTGTTGTTTA	G...	AGTC	ATAACTTCGTATAATGTATGCTATACGAAGTTAT	CACTAGTCAA
1	#19-079	ATTCTTAGGTCAGATGTAGTGTTGTTTA	GAC.	..TC	ATAACTTCGTATAATGTATGCTATACGAAGTTAT	CACTAGTCAA
1	#19-084	ATTCTTAGGTCAGATGTAGTGTTGTTTA	G...	AGTC	ATAACTTCGTATAATGTATGCTATACGAAGTTAT	CACTAGTCAA
1	#19-095	ATTCTTAGGTCAGATGTAGTGTTGTTTA	G...	AGTC	ATAACTTCGTATAATGTATGCTATACGAAGTTAT	CACTAGTCAA
1	#19-097	ATTCTTAGGTCAGATGTAGTGTT.....	....	....	ATAACTTCGTATAATGTATGCTATACGAAGTTAT	CACTAGTCAA
1	#19-098	ATTCTTAGGTCAGATGTAGTGTTGTTTA	G...	...C	ATAACTTCGTATAATGTATGCTATACGAAGTTAT	CACTAGTCAA
1	#19-099	ATTCTTAGGTCAGATGTAGTGTTGTTTA	G...	AGTC	ATAACTTCGTATAATGTATGCTATACGAAGTTAT	CACTAGTCAA
1	#19-101	ATTCTTAGGTCAGATGTAGTGTTGTTTA	G...	AGTC	ATAACTTCGTATAATGTATGCTATACGAAGTTAT	CACTAGTCAA
2	#19-065	ATTCTTAGGTCAGATGTAGTGTTGTTTA	GAC.	..TC	ATAACTTCGTATAATGTATGCTATACGAAGTTAT	CACTAGTCAA
2	#19-067	ATTCTTAGGTCAGATGTA..........	....	....	..AACTTCGTATAATGTATGCTATACGAAGTTAT	CACTAGTCAA
2	#19-072	ATTCTTAGGTCAGATGTAGTGTTGTTT.	....	AGTC	ATAACTTCGTATAATGTATGCTATACGAAGTTAT	CACTAGTCAA
2	#19-087	ATTCTTAGGTCAGATGTAGTGTTGTTTA	G...	AGTC	ATAACTTCGTATAATGTATGCTATACGAAGTTAT	CACTAGTCAA
2	#19-088	ATTCTTAGGTCAGATGTAGTGTTGTTTA	G...	AGTC	ATAACTTCGTATAATGTATGCTATACGAAGTTAT	CACTAGTCAA
2	#19-089	ATTCTTAGGTCAGATGTAGTGTTGTTTA	G...	AGTC	ATAACTTCGTATAATGTATGCTATACGAAGTTAT	CACTAGTCAA
2	#19-106	ATTCTTAGGTCAGATGTAGTGTTGTTTA	....	AGTC	ATAACTTCGTATAATGTATGCTATACGAAGTTAT	CACTAGTCAA

= truncated lox

= assignable to either end; suggests overlap of ends contributes to linking

S2) Target events from the Plasmid 1 with short LB, reverse orientation insertions, linkages at RB-LB junctions

27 bp border residue inside intron

MIDDLE LINKAGE: RB-LB junction

INSERT #	Sample #	I-At.Act7 5' --->	<-- (RB (post-cut))	<-- LB (post-cut)	I-At.Act7 3'
		TATGGAGTTTGAGGATTCTTCTCGCTTCTGTCGATCTCTCGCTGTTATTTTTGTTTTTTTCAGTGAAGTGCTG	G	CAGGATATA TTCAATTGTAAATGAAGTT	GTTTAGTTCGAAATGACTTCGTG
1	#19-060	TATGGAGTTTGAGGATTCTTCTCGCTTCTGTCGATCTCTCGCTGTTATT.........................	.	....CAATTGTAAATGAAGTT	GTTTAGTTCGAAATGACTTCGTG
1	#19-068	TATGGAGTTTGAGGATTCTTCTCGCTTCTGTCGATCTCTCGCTGTTATTTTTGTTTTTTTCAGTGAAGT....	.	....AATTGTAAATGAAGTT	GTTTAGTTCGAAATGACTTCGTG
1	#19-069	TATGGAGTTTGAGGATTCTTCTCGCTTCTGTCGATCTCTCGCTGTTATTTTTGTTTTTTTCAGTGAAGTGC..	.	....TAAATGAAGTT	GTTTAGTTCGAAATGACTTCGTG
1	#19-070	TATGGAGTTTGAGGATTCTTCTCGCCGCTGTCGATCTCTCGCT..............................	.	TCTGTCAA	...................TCGTG
1	#19-073	TATGGAGTTTGAGGATTCTTCTCGCTTCTGTCGATCTCTCGCTGTTATTTTTGTTTTTTTCAGTGAAGTGCTG	G	....TTATAT TCAATTGTAAATGAAGTT	GTTTAGTTCGAAATGACTTCGTG
1	#19-074	TATGGAGTTTGAGGATTCTTCTCGCTTCTGTCGATCTCTCGCTGTTATTTTTGTTTTTTTCAGT.........	.	....TAAATGAAGTT	GTTTAGTTCGAAATGACTTCGTG
1	#19-075	TATGGAGTTTGAGGATTCTTCTCGCTTCTGTCG..	.	....	..........AAATGACTTCGTG
1	#19-079	TATGGAGTTTGAGGATTCTTCTCGCTTCTGTCGATCTCTCGCTGTTATT.........................	.	.... .CAATTGTAAATGAAGTT	GTTTAGTTCGAAATGACTTCGTG
1	#19-084	TATGGAGTTTGAGGATTCTTCTCGCTTCTGTCGATCTCTCGCTGTTATTTTTGTTTTTTTCAGTGAAGTGC..	.	.........T TCAATTGTAAATGAAGTT	GTTTAGTTCGAAATGACTTCGTG
1	#19-095	TATGGAGTTTGAGGATTCTTCTCGCTTCTGTCGATCTCTCGCTGTTATTTTT.....................	.	....GTT	GTTTAGTTCGAAATGACTTCGTG
1	#19-097	TATGGAGTTTGAGGATTCTTCTCGCTTCTGTCGATCTCTCGCTGTTATTTTTGTTTTTTTCAGTGAAGTGCTG	G	ACCCCCG AT TCAATTGTAAATGAAGTT	GTTTAGTTCGAAATGACTTCGTG
1	#19-098	TATGGAGTTTGAGGATTCTTCTCGCTTCTGTCGATCTCTCGCTGTTATTTTTGTTTTTTTCAGTGAAGTGCT.	.	..GGATATAT TCAATTGTAAATGAAGTT	GTTTAGTTCGAAATGACTTCGTG
1	#19-099	TATGGAGTTTGAGGATTCTTCTCGCTTCTGTCGATCT.............................TCCAGCAAATCAAAATCAACATTCCTAGATTGCATCCC			..........ATGACTTCGTG
1	#19-101	TATGGAGTTTGAGGATTCTTCTCGCTTCTGTCGATCTCTCGCTGTTATTTTTGTTTTTTTCAGTGAAGTGC..	.	........AT TCAATTGTAAATGAAGTTG	TTTAGTTCGAAATGACTTCGTG
2	#19-065	TATGGAGTTTGAGGATTCTTCTCGCTTCTGTCGATCTCTCGCTGTTATTTTTGTTTTTTTCAGTGAAGTGCTG	G	. TAATTCTTATTTTTGTTTTTTTCAG .TGAAGTTG	TTTAGTTCGAAATGACTTCGTG
2	#19-067	TATGGAGTTTGAGGATTCTTCTCGCTTCTGTCGATCTCTCGCTGTTATTTTTGTTTTTTTCAGTGAAGTGCTG	G	....	.....................TG
2	#19-072	TATGGAGTTTGAGGATTCTTCTCGCTTCTGTCGATCTCTCGCTGTTATTTTTGTTTTTT..............	.	....ATTGTAAATGAAGTTG	TTTAGTTCGAAATGACTTCGTG
2	#19-087	TATGGAGTTTGAGGATTCTTCTCGCTTCTGTCGATCTCTCGCTGTTATTTTTGTTTTTTTCAGTGAAGTGCTG	G	....TATAT TCAATTGTAAATGAAGTTG	TTTAGTTCGAAATGACTTCGTG
2	#19-088	TATGGAGTTTGAGGATTCTTCTCGCTTCTGTCGATCTCTCGCTGTTATTTTTGTTTTTTTCA...........	.	....GTAAATGAAGTTG	TTTAGTTCGAAATGACTTCGTG
2	#19-089	TATGGAGTTTGAGGATTCTTCTCGCTTCTGTCGATCTCTCGCTGTTATTTTTGTTTTTTTCAGTGAA......	.	CTCACCCAGACGTCACTTAA ....TTG	TTTAGTTCGAAATGACTTCGTG
2	#19-106	TATGGAGTTTGAGGATTCTTCTCGCTTCTGTCGATCTCTCGCTGTTATTTTTGTTTTTTTCAGTGA.......	.	....AATTGTAAATGAAGTTG	TTTAGTTCGAAATGACTTCGTG

■ (red) = SNP

■ (pink) = overlap possible

■ (yellow) = insertion

Note: Filler DNAs are likely originated from host genomes or binary vectors. Thanks to an anonymous reviewer who has mapped two filler DNAs to soybean genome.
The filler DNA in the sample #19-99 has been mapped to *Glycine_max*_v2.0:3:1:45779434:1 REF, position: 40891130-40891166.
The filler DNA in the sample #19-089 has been mapped to *Glycine_max*_v2.0:11:1:34766867:1 REF, position: 29587578-29587597 and Glycine_max_v2.0:14:1:49042192:1 REF, position: 33094949-33094967 (reverse, complement), respectively.

S3) Target events from the Plasmid 1 with short LB, reverse orientation insertions, chromosome linkages at GOI terminator

T-DNA | <----Chr06

T-Mt.PSII---> | DT5.1 --> | <----DT5.1

INVERTED REPEAT----> | <-----INVERTED REPEAT

# INSERTS	Sample #	T-Mt.PSII		INVERTED REPEAT---->	<-----INVERTED REPEAT	
		TTAGCTTTTGTTTTTACTGTTGGTTTTTTGCCAAA	GCGGCCG	CTTTAGACTTTAGCTCCTCTGTTG	CAACAGAGGAGCTAAAGTCTAAAG	ACAGAACGAAGGAAAGAAATAATAAT
1	#19-060	TTAGCTTTTGTTTTTACTGTTGGTTTTTTGCCAAA	GCGGCCG	CTTTAGACT...............	......AGGAGCTAAAGTCTAAAG	ACAGAACGAAGGAAAGAAATAATAAT
1	#19-068	TTAGCTTTTGTTTTTACTGTTGGTTTTTTGCCAAA	GCGGCCG	CTTTA...................	.....................AAG	ACAGAACGAAGGAAAGAAATAATAAT
1	#19-069	TTAGCTTTTGTTTTTACTGTTGGTTTTTTGCCAAA	GCGGCCG	CTTTAGACTTTAGCTCCTC.....	....AGAGGAGCTAAAGTCTAAAG	ACAGAACGAAGGAAAGAAATAATAAT
1	#19-070	TTAGCTTTTGTTTTTACTGTTGGTTTTTTGCCAAA	GCGGCCG	CTTTAGACTTTAGCT.........	.. AAACACGAGCTTG ....TAAAG	ACAGAACGAAGGAAAGAAATAATAAT
1	#19-073	TTAGCTTTTGTTTTTACTGTTGGTTTTTTGCCAAA	GCGGCCG	CTTTAGACTTTAGCTCCTC.....	..........GCTAAAGTCTAAAG	ACAGAACGAAGGAAAGAAATAATAAT
1	#19-074	TTAGCTTTTGTTTTTACTGTTGGTTTTTTGCCAAA	GCGGCCG	CTTTAGACTTTAGCTCCTCT....	.............AAAGTCTAAAG	ACAGAACGAAGGAAAGAAATAATAAT
1	#19-075	TTAGCTTTTGTTTTTACTGTTGGTTTTTTGCCAAA	GCGGCCG	CTTTAGACTTTA............	.....................AAG	ACAGAACGAAGGAAAGAAATAATAAT
1	#19-079	TTAGCTTTTGTTTTTACTGTTGGTTTTTTGCCAAA	GCGGCCG	CTTTAGACTTTAGCTCCTC.....	GAGGAGCTAAAGTCTAAAG	ACAGAACGAAGGAAAGAAATAATAAT
1	#19-084	TTAGCTTTTGTTTTTACTGTTGGTTTTTTGCCAAA	GCGGCCG	CTTTAGACTTTAGCTCCTC.....	...CAGAGGAGCTAAAGTCTAAAG	ACAGAACGAAGGAAAGAAATAATAAT
1	#19-095	TTAGCTTTTGTTTTTACTGTTGGTTTTTTGCCAAA	GCGGCCG	CTT.....................	......AGGAGCTAAAGTCTAAAG	ACAGAACGAAGGAAAGAAATAATAAT
1	#19-097	TTAGCTTTTGTTTTTACTGTTGGTTTTTTGCCAAA	GCGGCCG	CTTTAGACTTTAGC..........	...CAGAGGAGCTAAAGTCTAAAG	ACAGAACGAAGGAAAGAAATAATAAT
1	#19-098	TTAGCTTTTGTTTTTACTGTTGGTTTTTTGCCAAA	GCGGCCG	CTTTAGACTTTAGCTCCTC.....	......AGGAGCTAAAGTCTAAAG	ACAGAACGAAGGAAAGAAATAATAAT
1	#19-099	TTAGCTTTTGTTTTTACTGTTGGTTTTTTGCCAAA	GCGGCCG	CTTTAGACTTTAGCTCCTCTG...	......AGGAGCTAAAGTCTAAAG	ACAGAACGAAGGAAAGAAATAATAAT
1	#19-101	TTAGCTTTTGTTTTTACTGTTGGTTTTTTGCCAAA	GCGGCCG	CTTTAGACTTTAGCTCCTC.....	......AGGAGCTAAAGTCTAAAG	ACAGAACGAAGGAAAGAAATAATAAT
2	#19-065	TTAGCTTTTGTTTTTACTGTTGGTTTTTTGCCAAA	GCGGCCG	CTTTAGACTT..............	.........AGCTAAAGTCTAAAG	ACAGAACGAAGGAAAGAAATAATAAT
2	#19-067	TTAGCTTTTGTTTTTACTGTTGGTTTTTTGCCAAA	GCGGCCG	CT......................	....................AAAG	ACAGAACGAAGGAAAGAAATAATAAT
2	#19-072	TTAGCTTTTGTTTTTACTGTTGGTTTTTTGCCAAA	GCGGCCG	CTTTAGACTTTAGCTCCTCT....	......AGGAGCTAAAGTCTAAAG	ACAGAACGAAGGAAAGAAATAATAAT
2	#19-087	TTAGCTTTTGTTTTTACTGTTGGTTTTTTGCCAAA	GCGGCCG	CTTTAGACTTTAGCTCC.......	....	..AGAACGAAGGAAAGAAATAATAAT
2	#19-088	TTAGCTTTTGTTTTTACTGTTGGTTTTTTGCCAAA	GCGGCCG	CTTTAGACTTTAGCTCCTC	GAGGAGCTAAAGTCTAAAG	ACAGAACGAAGGAAAGAAATAATAAT
2	#19-089	TTAGCTTTTGTTTTTACTGTTGGTTTTTTGCCAAA	GCGGCCG	CTTTAGAC................	......................AG	ACAGAACGAAGGAAAGAAATAATAAT
2	#19-106	TTAGCTTTTGTTTTTACTGTTGGTTTTTTGCCAAA	GCGGCCG	CTTTAGACTTTAGC..........	............TAAAGTCTAAAG	ACAGAACGAAGGAAAGAAATAATAAT

S4) Target events from the Plasmid 1 with short LB, forward orientation insertions, chromosome linkages at *lox* end of T-DNA

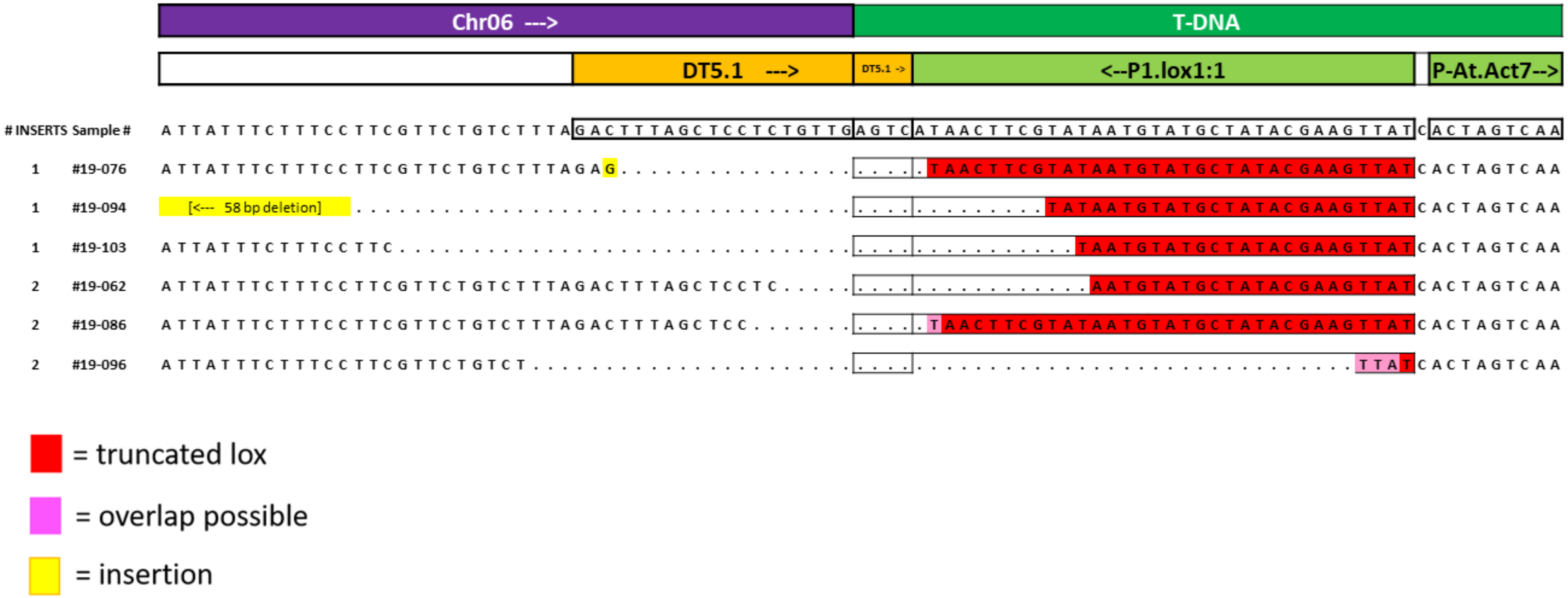

S5) Target events from the Plasmid 1 with short LB, forward orientation insertions, linkages at RB-LB junctions

27 bp border residue inside intron

# INSERTS	Sample #	I-At.Act7 5'---> / RB (post-cut) <--	<-- LB (post-cut) / I-At.Act7 3'
		TATGGAGTTTGAGGATTCTTCTCGCTTCTGTCGATCTCTCGCTGTTATTTTTGTTTTTTTCAGTGAAGTGCTGG	CAGGATATATTCAATTGTAAATGAAGTTGTTTAGTTCGAAATGACTTCGTG
1	#19-076	TATGGAGTTTGAGGATTCTTCTCGCTTCTGTCGATCTCTCGCTGTTATTTTTGTTTTTTTCAGTGAAGTGCTGG	TAAATGAAGTTGTTTAGTTCGAAATGACTTCGTG
1	#19-094	TATGGAGTTTGAGGATTCTTCTCGCTTCTGTCGATCTCTCGCTGTTATTTTTGTTTTTTTCAGT	AATGAAGTTGTTTAGTTCGAAATGACTTCGTG
1	#19-103	TATGGAGTTTGAGGATTCTTCTCGCTTCTGTCGATCTCTCGCT	TCTGAGC [--->145 bp deletion of LB] TAATC
2	#19-062	TATGGAGTTTGAGGATTCTTCTCGCTTCTGTCGATCTCTCGCTGTTATTTTTGTTTTTTTCAGTGAAGTGCTG	TTTAGTTCGAAATGACTTCGTG
2	#19-086	TATGGAGTTTGAGGATTCTTCTCGCTTCTGTCGATCTCTCGCT	GTTGTTTAGTTCGAAATGACTTCGTG
2	#19-096	TATGGAGTTTGAGGATTCTTCTCGCTTCTGTCGATCTCTCGCTGTTATTTTTGTTTTTTTCAGTGAAGTGCTGG	TGTAAATGGCGTTGTTTAGTTCGAAATGACTTCGTG

S6) Target events from the Plasmid 1 with short LB, forward orientation insertions, chromosome linkages at GOI terminator

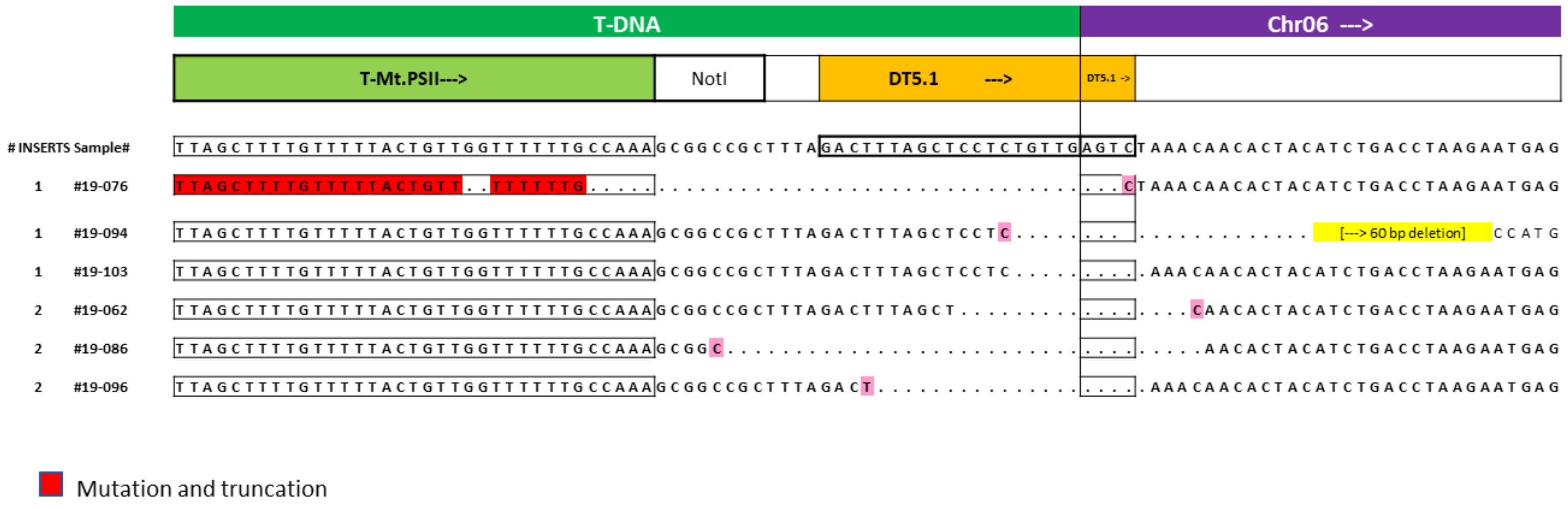

S7) Target events from the Plasmid 2 with long LB, reverse orientation insertions, chromosome linkages at *lox* end of T-DNA

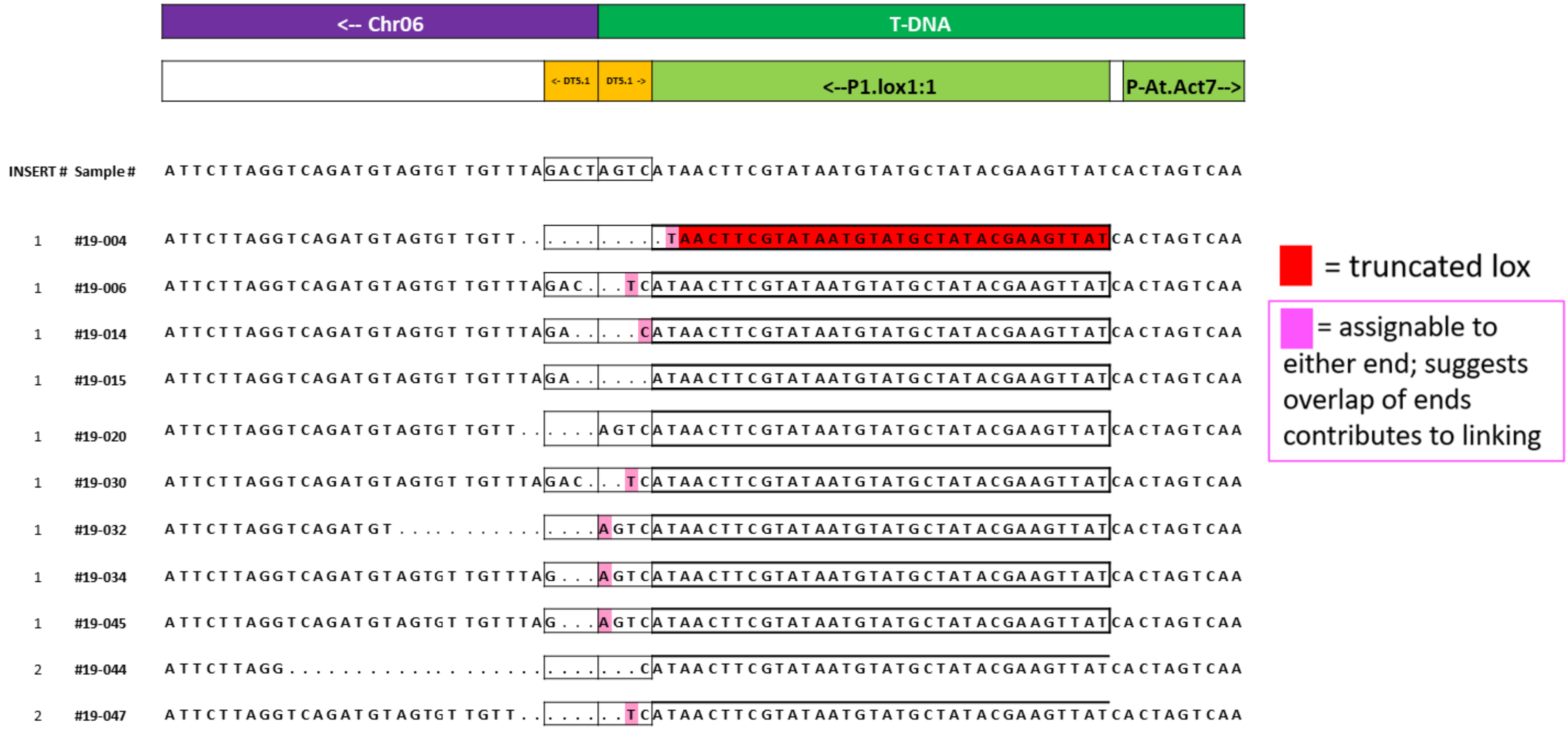

S8) Target events from the Plasmid 2 with long LB, reverse orientation insertions, linkages at RB-LB junctions

289 bp border residue inside intron

RB (post-cut)

INSERT #	Sample #	I-At.Act7 5' ---> / <--	<-- LB (post-cut)
		TATGGAGTTTGAGGATTCTTCTCGCTTCTGTCGATCTCTCGCTGTTATTTTTGTTTTTTTCAGTGAAGTGCTGG	CAGGATATATTCAATTGTAAATGGCTTCATGTCCGGGAAATCTACATGGAT
1	#19-004	TATGGAGTTTGAGGATTCTTCTCGCTTCTGTCGATCTCTCGCTGTTATTTTTGTTTTTTTCAGTGAAGTGCTGG	...AT
1	#19-006	TATGGAGTTTGAGGATTCTTCTCGCTTCTGTCGATCTCTCGCT...	...GCTTCATGTCCGGGAAATCTACATGGAT
1	#19-014	TATGGAGTTTGAGGATTCTTCTCGCTTCTGTCGATCTCTCGCTGTTATTTTTGTTTTTTTCAGTGAAGTG...	...TCCGGGAAATCTACATGGAT
1	#19-015	TATGGAGTTTGAGGATTCTTCTCGCTTCTGTCGATCTCTCGCTGTTATTTTTGTTTTTTTCAGTGAAGTGCTGG	... [--->338 bp deletion of LB, 7 bp unannotated insertion]
1	#19-020	TATGGAGTTTGAGGATTCTTCTCGCTTCTGTCGATCTCTCGCTGTTATTTTTGT...	...GGCTTCATGTCCGGGAAATCTACATGGAT
1	#19-030	TATGGAGTTTGAGGATTCTTCTCGCTTCTGTCGATCTCTCGCTGTTATTTTTGTTTTTTTCAGTGA...	...TCCGGGAAATCTACATGGAT
1	#19-032	T... [+10bp insetion] ...	...TATATTCAATTGTAAATGGCTTCATGTCCGGGAAATCTACATGGAT
1	#19-034	TATGGAGTTTGAGGATTCTTCTCGCTTCTGTCGATCTCTCGCTGTTATTTTTGTTTTTTTCAGTGAAGTGCTGG	...CTACATGGAT
1	#19-045	TATGGAGTTTGAGGATTCTTCTCGCTGTTAT...	... [---> 145 bp deletion of LB]
2	#19-044	TATGGAGTTTGAGGATTCTTCTCGCTTCTGTCGATCTCTCGCTGTTATTTTTGTTTTTTTCAGTGAAGTGC...	... [--->66 bp deletion of LB]
2	#19-047	TATGGAGTTTGAGGATTCTTCTCGCTTCTGTCGATCTCTCGCTGTTATTTTTGTTTTT...	... [--->113 bp deletion of LB]

S9) Target events from the Plasmid 2 with long LB, reverse orientation insertions, chromosome linkages at GOI terminator

T-DNA | <-----Chr06

T-Mt.PSII---> | DT5.1 --> | <----DT5.1

INVERTED REPEAT----> | <-----INVERTED REPEAT

INSERT #	Sample#	T-Mt.PSII--->		INVERTED REPEAT---->	<-----INVERTED REPEAT	
		TTAGCTTTTGTTTTTACTGTTGGTTTTTTGCCAAA	GCGGCCG	CTTTAGACTTTAGCTCCTCTGTTG	CAACAGAGGAGCTAAAGTCTAAAG	ACAGAACGAAGGAAAGAAATAATAAT
1	#19-004	TTAGCTTTTGTTTTTACTGTTGGTTTTTTGCCAAA	GCGGCCG	CTTTAGACTTTAGCTCCTC.....	...............AGTCTAAAG	ACAGAACGAAGGAAAGAAATAATAAT
1	#19-006	TTAGCTTTTGTTTTTACTGTTGGTTTTTTGCCAAA	GCGGCCG	CTTTAGACTTTAGCTCCTC.....	.....GAGGAGCTAAAGTCTAAAG	ACAGAACGAAGGAAAGAAATAATAAT
1	#19-014	TTAGCTTTTGTTTTTACTGTTGGTTTTTTGCCAAA	GCGGCCG	CTTTAGACTTT.............	.............AAAGTCTAAAG	ACAGAACGAAGGAAAGAAATAATAAT
1	#19-015	TTAGCTT............................	....	....	....................AAAG	ACAGAACGAAGGAAAGAAATAATAAT
1	#19-020	TTAGCTTTTGTTTTTACTGTTGGTTTTTTGCCAAA	GCGGCCG	CTTTAG..................	........GAGCTAAAGTCTAAAG	ACAGAACGAAGGAAAGAAATAATAAT
1	#19-030	TTAGCTTTTGTTTTTACTGTTGGTTTTTTGCCAAA	GCGGCCG	CTTTAGACTTTAGCTCCTC.....	...........CTAAAGTCTAAAG	ACAGAACGAAGGAAAGAAATAATAAT
1	#19-032	TTAGCTTTTGTTTTTACTGTTGGTTTTTTGCCAAA	GCGGCCG	CTTTAG..................	..................CTAAAG	ACAGAACGAAGGAAAGAAATAATAAT
1	#19-034	TTAGCTTTTGTTTTTACTGTTGGTTTTTTGCCAAA	GCGGCCG	CTTTAGA.................	....	.................AATAATAAT
1	#19-045	TTAGCTTTTGTTTTTACTGTTGGTTTTTTGCCAAA	GCGGCCG	CTTTAGACTTTAGCTCCT......	...CAGAGGAGCTAAAGTCTAAAG	ACAGAACGAAGGAAAGAAATAATAAT
2	#19-044	TTAGCTTTTGTTTTTACTGTTGGTTTTTTGCCAAA	GCGGCCG	CTTT....................	......................AG	ACAGAACGAAGGAAAGAAATAATAAT
2	#19-047	TTAGCTTTTGTTTTTACTGTTGGTTTTTTGCCAAA	GCGGCCG	CTTTAGACTTTAGCTCCTC.....	......AGGAGCTAAAGTCTAAAG	ACAGAACGAAGGAAAGAAATAATAAT

S10) Target events from the Plasmid 2 with short LB, forward orientation insertions, chromosome linkages at *lox* end of T-DNA

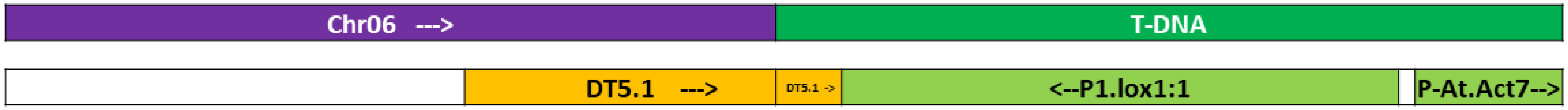

INSERT#	Sample #	Chr06 / DT5.1	DT5.1	<--P1.lox1:1	P-At.Act7
		ATTATTTCTTTCCTTCGTTCTGTCTTTAGACTTTAGCTCCTCTGTTG	AGTC	ATAACTTCGTATAATGTATGCTATACGAAGTTAT	CACTAGTCAA
2	#19-029	ATTATTTCTTTCCTT................................	....	......TTCGTATAATGTATGCTATACGAAGTTAT	CACTAGTCAA
2	#19-035	ATTATTTCTTTCCTTCGTTCTGTCTTTAGACTTTAG......CA...	AGTC	ATAACTTCGTATAATGTATGCTATACGAAGTTAT	CACTAGTCAA

Red = truncated lox

Yellow = insertion

S11) Target events from the Plasmid 2 with long LB, forward orientation insertions, linkages at RB-LB junctions

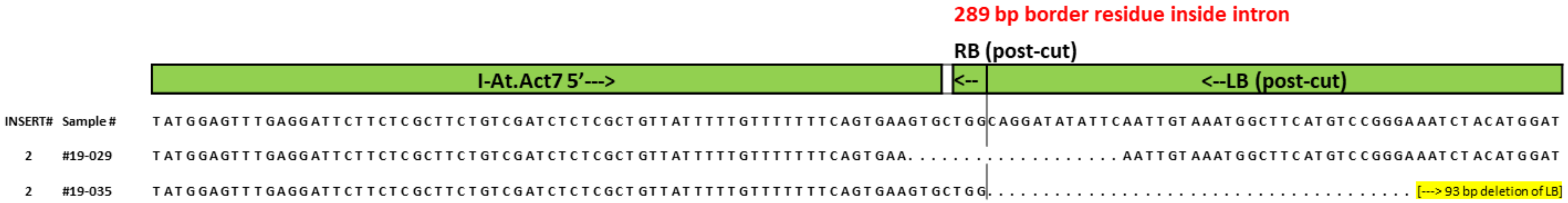

S12) Target events from the Plasmid 2 with long LB, forward orientation insertions, chromosome linkages at GOI terminator

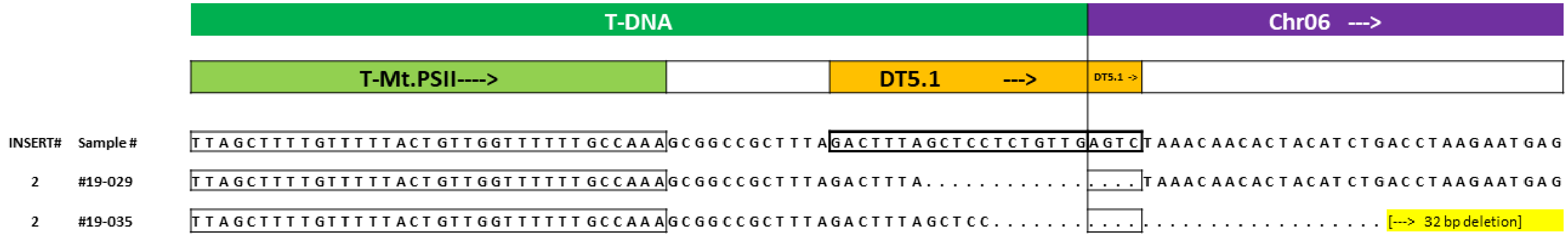

Supplement: Supplementary file 1 — Appendix S1: Materials and Methods; Supplementary 1‐4 for genetic elements, Table S1‐S6, Supplemental Figures, and detailed NHEJ junction analyses. [file PBI-22-1926-s001.pdf]
